# Nuclear Receptor-Mediated Metabolic Reprogramming and the Impact on HR+ Breast Cancer

**DOI:** 10.3390/cancers13194808

**Published:** 2021-09-26

**Authors:** Shaimaa Hussein, Pooja Khanna, Neha Yunus, Michael L. Gatza

**Affiliations:** 1 Rutgers Cancer Institute of New Jersey, New Brunswick, NJ 08903, USA; sh1463@cinj.rutgers.edu (S.H.); poojak@cinj.rutgers.edu (P.K.); 2 Department of Radiation Oncology, Robert Wood Johnson Medical School, Rutgers, The State University of New Jersey, New Brunswick, NJ 08903, USA; 3 School of Arts and Sciences, Rutgers, The State University of New Jersey, New Brunswick, NJ 08903, USA; nny7@scarletmail.rutgers.edu

**Keywords:** estrogen receptor, breast cancer, nuclear receptors, metabolic reprogramming, endocrine resistance, luminal

## Abstract

**Simple Summary:**

Breast cancer is the most commonly diagnosed and second leading cause of cancer-related deaths in women in the United States, with hormone receptor positive (HR+) tumors representing more than two-thirds of new cases. Recent evidence has indicated that dysregulation of multiple metabolic programs, which can be driven through nuclear receptor activity, is essential for tumor genesis, progression, therapeutic resistance and metastasis. This study will review the current advances in our understanding of the impact and implication of altered metabolic processes driven by nuclear receptors, including hormone-dependent signaling, on HR+ breast cancer.

**Abstract:**

Metabolic reprogramming enables cancer cells to adapt to the changing microenvironment in order to maintain metabolic energy and to provide the necessary biological macromolecules required for cell growth and tumor progression. While changes in tumor metabolism have been long recognized as a hallmark of cancer, recent advances have begun to delineate the mechanisms that modulate metabolic pathways and the consequence of altered signaling on tumorigenesis. This is particularly evident in hormone receptor positive (HR+) breast cancers which account for approximately 70% of breast cancer cases. Emerging evidence indicates that HR+ breast tumors are dependent on multiple metabolic processes for tumor progression, metastasis, and therapeutic resistance and that changes in metabolic programs are driven, in part, by a number of key nuclear receptors including hormone-dependent signaling. In this review, we discuss the mechanisms and impact of hormone receptor mediated metabolic reprogramming on HR+ breast cancer genesis and progression as well as the therapeutic implications of these metabolic processes in this disease.

## 1. Introduction

Globally, female breast cancer is the most commonly diagnosed form of cancer and is the fifth leading cause of cancer-related deaths [1]. In the United States, despite advances in treatments and the implementation of preventive care measures, including early screening, breast cancer remains the most frequently diagnosed and second leading cause of cancer-related deaths accounting for more than 270,000 new cases and more than 42,000 estimated deaths in 2020 [2]. Pathological biomarkers, including estrogen receptor (ER), progesterone receptor (PR) and HER2 (human epidermal growth factor receptor 2) provide the clinical basis for classifying breast tumors and directing therapeutic strategies. Hormone receptor positive (HR+) tumors which express ER and PR represent 60–70% of new cases, HER2+ tumors represent 10–20% of cases, while the remaining 15–20% of tumors are defined as triple negative breast cancers (TNBC) and lack expression of ER, PR and HER2. It should be noted that differences in frequencies of clinical subtypes are evident between patients of different racial ancestries and between different countries; we point our readers to a number of excellent review articles that discuss the recent advances in our understanding of these differences [3,4,5,6]. At the molecular level, distinct disease subtypes have been identified (i.e., basal-like, Luminal A, Luminal B, HER2E) and are characterized by unique patterns of genomic and proteomic alterations that translate into altered cellular signaling, tumor metabolism, and therapeutic response [7,8,9,10,11,12,13,14,15]. Owing in part to the dependency of HR+ tumors on endocrine signaling, hormone-based treatments including aromatase inhibitors, selective estrogen receptor modulators (SERM), and selective estrogen receptor degraders (SERD) provide the basis for treatment of this disease [16,17,18].

Hormone receptors (HRs), including ER and PR, belong to an evolutionarily conserved family of nuclear receptors that are responsible for regulating a multiple cellular and systemic biological processes required for normal breast development and, when aberrantly activated, contribute to tumorigenesis [19,20,21]. These receptors modulate cellular activity through both ligand-dependent and non-canonical ligand independent activity [22]. In HR+ breast cancer, both ER and PR are commonly co-expressed due, in part, to ER-dependent activation of PR expression [23,24]. These receptors play a prominent role in regulating cellular growth and tumor progression through activation of kinase signaling cascades and transcriptional regulation of signaling networks [25,26,27,28,29,30]. ER mediates effects on cellular signaling through ligand (estradiol)-induced activation of kinase signaling cascades resulting in aberrant PI3K/AKT/mTOR [25,26,31], Notch [27,28,32,33,34] and RAS/MAPK [29,30,35,36] signaling. Following receptor internalization and nuclear translocation, ER functions as a transcription factor by binding to estrogen response elements (EREs) to mediate expression of genes that promote cell proliferation and survival [37,38,39,40]. Importantly, loss of ER expression and estrogen or ligand-dependent growth leads to increased tumor aggressiveness and decreased therapeutic response. Ligand independent ERα activation and estrogen-independent cell growth can be due to *ESR1* mutations, altered MAPK signaling, MYC or transcription factor activation, and/or activation of key growth factors including HER2 and FGFR1 [41,42].

Similar to ER, PR acts in a ligand-dependent manner to regulate transcriptional activity through direct binding to progesterone response elements (PRE) [43]. A number of studies have indicated that progesterone limits breast tumor growth through ER-dependent and independent mechanisms [44,45,46,47,48]. Notably, PR has been shown to cooperate with ERα to regulate expression of an anti-proliferative gene expression profile associated with a good prognosis, while PR alone can mitigate cell proliferation through several mechanisms including repression of cyclin dependent kinases and MAPK signaling [44,45,46,47,48]. Not surprisingly, PR expression serves as a predictive parameter for endocrine therapy response [47]. In many cases, as HR+ tumors progress, many lose ER and/or PR expression and, as such, loss of PR expression is associated with a significantly worse prognosis [47,49]. Several studies have indicated that PR loss contributes to an aggressive phenotype as a result of activating a transcriptomic profile which includes increased EGFR and NOTCH1 signaling as well as expression of genes that regulate amino acid uptake. This expression profile, which is similar to profiles expressed by aggressive basal-like tumors, can be induced by PR loss irrespective of ER status [50,51,52].

In addition to ER and PR, a number of other nuclear receptors are involved in promoting tumorigenesis in HR+ breast cancer. Among these, androgen receptor (AR) is a ligand activated hormone receptor with transcriptional activities involved in cell proliferation and is expressed in both TNBC and HR+ tumors [53]. AR has been shown to play a dichotomous role in HR+ breast cancer. In these tumors increased AR expression corresponds with a favorable prognosis by serving as an ER antagonist [54,55]. However, additional studies have reported that when constitutively activated through elevated steroid ligand levels, high AR expression can contribute to transcriptional changes that promote endocrine therapy resistance [55]. Other hormone nuclear receptors including glucocorticoid receptors (GR) [56] and retinoic acid receptors (RAR) as well as adopted nuclear receptors including peroxisome proliferative activated receptors (PPAR), retinoid x receptors (RXR), estrogen-related receptors (ERRs) and liver x receptors (LXRs) have also been implicated in a various aspects of breast cancer tumor progression including the regulation of apoptotic and proliferative signaling [19,57,58]. However, further research is still required to better understand their exact role and therapeutic potential.

In addition to the noted effects of hormone receptors on the oncogenic signaling pathway in tumor development and progression, emerging data suggest that these receptors can also modulate effects on tumor metabolism and metabolic reprogramming in HR+ breast cancer [19,20,21,59]. Based on seminal studies by Warburg, tumor metabolism has been acknowledged as a key hallmark of cancer since the early 1900s [60]. Cancer cells have the ability to alter activated metabolic pathways in order to adapt to the changing microenvironment and/or to available resources [61,62]. In HR+ breast cancer, nuclear receptors, including ER have been shown to directly regulate metabolic reprogramming by affecting the expression of key metabolic genes and indirectly by modulating the expression and activity of oncogenes and oncogenic signaling pathways (Table 1) [19,20,21,59].

**Figure 1:**
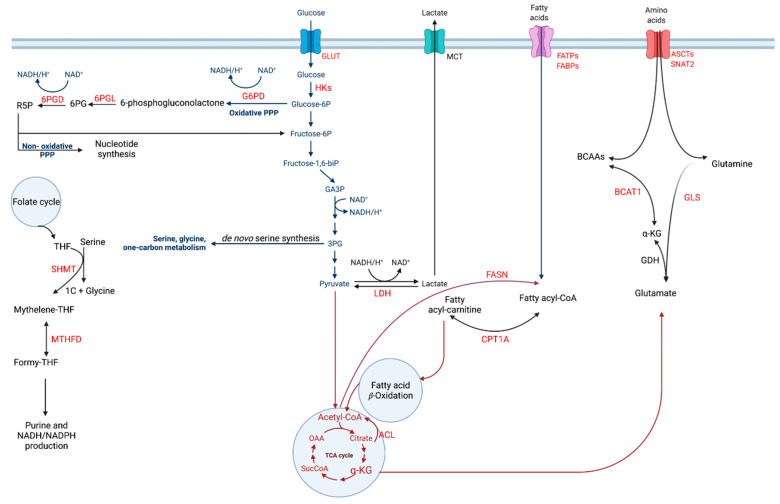
Schematic representation of the crosstalk between cancer-associated major metabolic pathways altered in HR+ breast cancer.

In this review, we will discuss the major metabolic pathways directly or indirectly regulated by nuclear receptor activity in HR+ breast cancer including glucose metabolism, amino acid metabolism, and fatty acid metabolism (summarized in Figure 1); recent advances in our understanding of the role of nuclear receptors in modulating these pathways; and the role of these metabolic processes in tumorigenesis, including therapeutic response.

## 2. Glucose Metabolism

### 2.1. Glycolysis

Cells conventionally funnel glucose through the tricarboxylic acid (TCA) cycle to generate ATP in the presence of oxygen [63]. In contrast to normal cells, cancer cells have increased rates of aerobic glycolysis, glutaminolysis and fatty acid synthesis [64]. The use of these pathways is essential for rapid generation of ATP and other metabolites which enable cancer cells to meet their energy needs in order to maintain increased cell proliferation and tumor growth [64]. Metabolic intermediates from the glycolysis pathway can be further used for fatty acid, nucleic acid and amino acid synthesis [63,64].

In HR+ breast cancer, ER and PR activity have been reported to affect the glycolytic capacity of cells through transactivation of key members of the glycolysis pathway. The initial step in glycolysis is regulated by glucose transport (GLUT) proteins which facilitate the transport of glucose across the cellular membrane [65,66,67,68]. While a number of studies suggest that GLUT1 is the predominant glucose transporter expressed in these cells, HR+ breast tumors demonstrate increased expression or translocation of additional GLUT proteins, including GLUTs 1—5, while GLUT12 has been detected at higher levels in ductal cell carcinoma in situ samples [65,66,67,68]. Mechanistically, ER and estrogen-dependent signaling have been shown to promote expression of GLUT proteins, notably GLUT1 and GLUT4, in order to increase glucose uptake and glycolysis. Likewise, progesterone alone, and in combination with estradiol, has been reported to upregulate GLUT1, GLUT3 and GLUT4 expression on a transcriptional level [68]. However, the impact of estrogen signaling on specific GLUT proteins remains unresolved and is likely dependent on cellular context as conflicting reports have been published outlining the impact of estrogen signaling on these proteins in different breast cancer cell line and tumor models [65,66,67,69,70,71,72].

Beyond regulation of GLUT protein transcription, ER and PR can affect the glycolytic capacity of cells through transactivation of key members of the glycolysis pathway including LDHA which catalyzes the reversible conversion of pyruvate to lactate and PKM2 which catalyzes the phosphoenolpyruvate (PEP) to pyruvate conversion. In addition, ER/PR activity can indirectly promote glycolysis through activation of PI3K and EGFR signaling [69,70]. Collectively, these findings suggest that ER or estrogen, and to a lesser extent PR, can promote glycolysis in ER+ cells in order to promote tumorigenesis; however, the precise mechanism(s), the role of specific co-factors present in distinct model systems, and the impact of ER/PR-driven glycolysis in ER+ breast cancer tumorigenesis remain unclear.

### 2.2. The Pentose Phosphate Pathway (PPP)

Glucose is converted to glucose-6-phosphate (G6P) by the hexokinase (HK) enzyme during the first stages of glycolysis and is then shunted into the pentose phosphate pathway (PPP) (Figure 1) which is essential for nucleotide synthesis [73]. During the first phase of PPP, G6P is converted to 6-phosphogluconolactone by glucose 6-phosphate dehydrogenase (G6PD) which results in the production of NADPH. 6-Phosphogluconolactone is hydrolyzed by 6-phosphogluconolactonase (6PGL) to produce 6-phosphogluconate (6PG) resulting in the generation of the nucleic acid synthesis precursor ribulose 5-phosphate and the production of additional NADPH molecules by 6-phosphogluconate dehydrogenase (6PGD) in the last, and irreversible step of the oxidative phase of the PPP [74]. The final, non-oxidative reversible phase in this pathway generates ribose 5-phosphate by the action of ribose 5-phosphate isomerase (RPI) or xylulose 5-phosphate (Xu5P) by ribulose 5-phosphate epimerase (RPE) [73]. With the action of the enzymes transketolase and transaldolases, PPP is capable of recycling the resulting products back to the PPP oxidative phase where it utilizes G6P and glycolysis [75].

In contrast to glycolysis, PPP does not provide energy to the cells, but instead provides ribose 5-phosphate (R5P) for nucleotide synthesis. It is also the major source of NADPH necessary for fatty acids and sterols synthesis, antioxidant activity and amino acid production [74,75]. A number of studies have reported that genes involved in PPP are dysregulated in HR+ breast cancer and that altered expression of these genes by estrogen and/or estrogen-dependent signaling can directly or indirectly contribute to ER+ breast cancer tumorigenesis and progression [74,76]. Notably, G6PD, which is the rate limiting enzyme of the oxidative phase of PPP, has been shown to be aberrantly activated in HR+ breast cancer and contributes to tumor development and progression through decreased cellular autophagy, tumor relapse and drug resistance in breast cancer models. In these tumors, estrogen and ER-dependent signaling contribute to PPP by directly modulating G6PD and lactate dehydrogenase (LDH) expression and by indirectly mediating expression of PPP genes through altered PI3K/AKT, KRAS, cMYC or p53 signaling [77,78,79,80,81,82]. Cancer cells can dynamically switch to PPP which results in altered expression of key enzymes required to maintain stemness, overcome oxidative stress, and enhance dissemination capacity [83,84,85]. Consistent with the dependency of ER+ cell lines on PPP, G6PD inhibition can promote cell death through the unfolded protein response [86]. In summary, estrogen and ER regulate glucose metabolic pathways through different genetic targets and contribute to metabolic plasticity observed in the HR+ breast tumors enhancing tumor progression and ability to metastasize. This highlights the therapeutic potential of this pathway especially in the resistance state as we will discuss in Section 5.

## 3. Amino Acid Metabolism

### 3.1. Glutamine Metabolism

A generally non-essential amino acid under normal physiological growth conditions, glutamine has been shown to be essential for cancer progression [87,88,89,90,91,92,93]. Glutaminase (GLS) enzyme converts glutamine to glutamate in the first step of glutamine catabolism [94,95]. During tumorigenesis, glutamine contributes to nucleotide biosynthesis through the trifunctional carbamylphosphate synthetase/aspartate carbamyltransferase/dihydroorotase (CAD) enzyme [96,97]. Glutamine plays a further role in promoting glutathione production and amino acid synthesis through glutamate cysteine ligase (GCL) and glutamic-oxaloacetic transaminase (GOT), respectively [98,99]. In cancer cells, CAD converts glutamate to dihydroorotate [100], which can be converted to orotate by the mitochondrial enzyme, dihydroorotate dehydrogenase (DHODH) under normoxic conditions [101]. Alternatively, under hypoxic conditions, when glutamine metabolism is elevated, dihydroorotate can be secreted by the cancer cells to alleviate the resulting intracellular nitrogen burden [102].

In HR+ breast cancer, glutamine metabolism increases as a result of glutamine addiction and has been linked to tumor aggressiveness and resistance to endocrine therapy [95]. Consistent with these findings, ER-dependent expression of glutamine metabolism related genes has been reported. The strongest correlation has been shown to exist between ER status and the expression of 4-aminobutyrate aminotransferase (ABAT), an enzyme involved in the catabolism of gamma-aminobutyric acid (GABA) [103,104]. In addition, recent studies by Demas et al. have identified a link between the expression of other glutamine and/or glutamate transporter proteins and estrogen sensitivity [95]. This study reported that inhibiting expression of the glutamine transport protein ASCT2 in estrogen sensitive breast cancer cells resulted in a concomitant decrease in cell proliferation and decreased expression of the glutamine transport protein SNAT1 and glutamate transport protein EAAT2. Interestingly, however, the investigators were able to demonstrate that in ER+ cells that have acquired estrogen resistance, expression of SNAT1 and EAAT2 was maintained irrespective of ASCT2 expression and that glutamine dependency developed in an mTOR-dependent manner. Further studies have indicated that poor prognosis and glutamine dependency in breast cancer are associated with aberrant GLS expression [95]. GLS1 is the most commonly expressed glutaminase isoform in breast cancer cells; however, GLS2 has also been recently linked to sustained cell proliferation and tumorigenesis, including EMT and metastasis [105]. While both isoforms are transcriptionally activated in an ER-dependent manner, expression of the GLS isoforms are subtype-dependent as GLS1 is more prevalent in TNBC tumors while GLS2, which is transcriptionally regulated by the ER co-factor GATA3, is more common in HR+ tumors [105,106].

Consistent with evidence that ER can mediate glutamine metabolism in HR+ breast cancer, a number of studies have reported that ERα and/or other commonly activated oncogenes in ER+ breast cancer, including MYC, can directly or indirectly induce expression of CAD [107]. Conversely, the hypoxia response factor HIFα inhibits expression of this enzyme under hypoxic conditions [108,109]. In these studies, CAD expression, but not GOT, was reported to be essential for HR+ tumor cell growth under normal oxygen conditions. Additional studies have indicated that HIF1α down-regulation of CAD and GOT enzymes can promote ER+ tumor cell proliferation, which indicates that additional mechanism(s) may exist to compensate for loss of CAD activity under hypoxic conditions [102]. Finally, Hoffman et al. established that expression of DHODH is significantly associated with increased breast cancer risk [110]. In HR+ breast cancer, aberrant DHODH overexpression promotes cell proliferation and the use of DHODH inhibitors resulted in cell cycle arrest mediated by upregulation of p53, p65 and STAT6 proteins [111]. Despite these findings, the impact of ER and other nuclear receptors on DHODH expression and function remains unclear.

### 3.2. Branched-Chain Amino Acids (BCAAs) Metabolism

In addition to glutamine, a number of other amino acids, namely valine, leucine, and isoleucine are required for protein synthesis. Breast tumors are characterized by increased BCAA levels compared to adjacent normal tissue [112,113]. Interestingly, however, only leucine levels are reported to be positively associated with breast cancer risk in obese and postmenopausal women [114]. BCAAs are catabolized by the action of two groups of enzymes. The branched-chain-amino-acid aminotransferases (BCAT1 and BCAT2) control the reversible transfer of the amino group from the BCAAs to α-ketoglutarate (α-KG) whereas branched-chain α-keto acid dehydrogenase (BCKD), which is localized to the mitochondria, controls the irreversible transformation of branched-chain keto acids (BCKA) to their corresponding BC-acyl-CoA esters [115].

BCAT1 has been reported to be overexpressed in a number of forms of cancer; however, its role remains unclear as it appears to promote tumorigenesis and is required to maintain cell growth in some forms of cancer including gastric, endometrial and gliomas whereas in pancreatic and ovarian tumors, this gene is overexpressed but is not essential for viability [115,116,117,118,119,120]. Within the context of breast cancer, subtype specific expression of BCAT enzymes has been observed with the cytosolic isoform BCAT1 commonly expressed in breast tumors that lack estrogen receptor or ER-activity, including progressive HR+ tumors that have lost estrogen dependency [121,122]. Regardless, high BCAT1 expression corresponds with increased tumor aggressiveness and has been reported to be a potential prognostic marker in TNBC breast cancer as well as other malignancies including colorectal cancer [123,124]. In HR+ breast cancer cell lines, activation of BCAT1 activity through mTOR signaling has been shown to promote cell growth in vitro by regulating mitochondrial function and biogenesis [113]. Conversely, mitochondrial BCAT2, which is commonly expressed in ER+ tumors, has been shown to regulate isocitrate dehydrogenase 1 (IDH1) expression and is associated with good prognosis in breast cancer [121,122]. However, it remains to be determined specifically how ER and/or estrogen-dependent signaling regulate BCAA metabolism and whether endocrine-resistance or loss of ER-dependent cell growth promotes BCAT1 expression or if increased BCAT1 expression contributes to loss of estrogen-dependent cell growth. In addition to BCAT activity, HR+ breast tumors are characterized by increased expression of the leucine transporter, SLC7A5 which partners with the ER-regulated scaffolding protein, LLGL2, to promote tumor growth and tamoxifen resistance through metabolic remodeling that allows increased leucine uptake [125]. Although emerging data suggest that aberrant BCAA metabolism is present in some HR+ breast tumors, the exact impact of the altered signaling and the mechanisms regulating this metabolic pathway in these tumors remains unclear.

### 3.3. Serine Amino Acid Cycle and the Mitochondrial One-Carbon (1C) Metabolism

Over the past 10 years, significant progress has been made in our understanding of how cancer cells use one-carbon metabolism to synthesize anabolic precursors for nucleotide synthesis and for the production of methyl groups used for RNA, DNA and protein methylation, which collectively contribute to tumor growth [126,127]. Several studies have demonstrated that amino acid metabolic pathways, including serine and glycine, are connected to the folate cycle which provides the universal 1C acceptor tetrahydrofolate (THF). This intermediate metabolite can accept or donate the one-carbon units necessary to facilitate nucleotide synthesis and to provide methyl groups. In addition to THF, serine hydroxymethyltransferase 1 (SHMT1) and SHMT2, which are localized to the cytoplasm and mitochondria, respectively, are essential for catabolizing serine to glycine and 1C units [126,127]. Alternatively, methylenetetrahydrofolate dehydrogenase (MTHFD) which exists in both cytosolic (MTHFD1) and mitochondrial (MTHFD2, MTHFD1L, MTHFD2L) forms is required to convert methylene-THF to formyl-THF which is essential for purine biosynthesis and NADH/NADPH production [128,129,130,131,132].

Metabolic reprogramming enables cancer cells to switch to the de novo serine synthesis pathway in the case of serine dietary shortage by utilizing the glycolysis intermediate 3-phosphoglycerate (3-PG). Alternatively, cells can use external serine through autophagy or lysosomal metabolites to obtain sufficient serine to meet their demands [127,131,133,134]. While the exact role of serine methyltransferases in ER+ breast cancer remains unknown, a number of recent studies have begun to demonstrate a significant role for one carbon metabolism and SHMT2, which is transcriptionally activated by ERRα [133,135]. Notably, it has been reported that SHMT2 expression, and the one carbon metabolism pathway, is essential for metastasis in TNBC breast cancer models and correlates with poor clinical outcome in patients; however, the mechanisms by which this pathway promotes metastasis remain unclear [127,133]. Given that the antidepressant drug sertraline can inhibit the growth of 1C addicted breast cancer in xenograft cancer models, studies by the De Keersmaecker lab provide evidence for this pathway as a potential therapeutic target in these tumors [134]; however, the feasibility of this strategy in patients remains to be determined.

ER and other nuclear receptors play a role in facilitating amino acid metabolism and have been shown to contribute to the metabolic reprogramming essential for cell migration and metastatic capacity. Vazquez Rodriguez et al. showed the estrogen-mediated lysine-dependent upregulation of the 35 kDa subunit of the U2AF splicing factor (*U2AF1*) and ribophorin-2 (*RPN2*) increased the neutrophil-mediated dissemination ability of the ER+ breast cancer cells via increased cell proliferation, migration and expression of cell adhesion genes [136]. ERα has been shown to interact with the peroxisome proliferator-activated receptor gamma coactivator 1-alpha (*PPARGC1A)*, a transcriptional coactivator for many steroid hormone receptor and nuclear receptors, to allow Luminal B subtype HR+ breast cancer brain metastatic cells to grow independent of glucose. In doing so, these tumors become dependent on amino acid metabolism and this shift in metabolic state has been associated with poor prognosis [137]. Further studies have demonstrated that PGC1α can also interact with ERRα to maintain metabolic plasticity by promoting specific gene expression programs. This enables cancer cells, specifically invasive, metastatic and circulating tumor cells, to fulfill the high energy demand necessary to maintain growth under limited nutrient conditions [138,139,140,141,142].

## 4. Fatty Acid Metabolism

Fatty acids (FAs) represent one of the basic constituents of the cell. These molecules are required for the formation of the cellular membranes, play a pivotal role in cellular structure, serve as precursors for signaling molecules, and are essential for cellular metabolism in both normal and malignant cells [143,144]. Cancer cells have the ability to make their own fatty acids through fatty acid synthesis (FAS) or can acquire fatty acids secreted into the microenvironment by cancer associated fibroblasts (CAF) and adipose tissue [145,146]. To obtain energy, fatty acids undergo catabolic breakdown by fatty acid oxidation (FAO) in the mitochondria [147]. Reprogramming of fatty acid metabolism and differential expression of fatty acid metabolism genes between TNBC and HR+ breast cancer is profound and suggests that unique mechanisms are employed by each malignancy to regulate these molecules [148].

Beyond FAS and FAO, which will be discussed in greater detail below, cholesterol and neutral lipid metabolism reprogramming is evident in some HR+ breast cancers [149]. The nuclear receptor, liver X receptor (LXR), is known to regulate the expression of cholesterol efflux transporter proteins (e.g., ABCA1 and ABCG1). Breast tumors with high LXR expression are often characterized by increased neutral lipids and free cholesterol accumulation which has also been observed in tamoxifen resistant clones [150]. However, while these tumors tend to show increased expression of the immature form of the lysosomal protein cathepsin D, which results in altered lysosomal membrane permeabilization, the impact of LXR expression in HR+ tumors remain unclear. Recent studies have reported that LXR expression reduced the proliferative capacity of estrogen dependent HR+ cell lines in vitro [151,152]; however, this reduction was not evident in breast cancer mouse models where high levels of circulating cholesterol induced ER-dependent tumor growth and LXR-dependent metastasis [153]. While these results are seemingly in contradiction, a number of recent studies have begun to illuminate cellular and environmental cues that may contribute to these phenotypes. Recent work by Baek and colleagues suggests that 27-hydroxycholesterol is required to modulate ERα signaling in murine breast tumors whereas LXR-dependent metastasis requires the presence of neutrophils [154]. These results are consistent with additional studies that demonstrate that that exogenous cholesterol can promote metabolic reprograming in breast cancer cells through ERRα activity [155]. These cells demonstrate increased proliferation due to increased levels of OXPHOS, NADPH and TCA intermediates and are characterized by an ERRα gene expression signature which is similar to that observed in breast cancer patients who are obese or who have high cholesterol [155]. We will note that limited data have identified direct links between hormone receptor activity and regulation of downstream targets of neutral lipid metabolism in HR+ breast cancer. However, given the link between BMI and obesity with increased breast cancer risk and poor clinical outcome, this is a complex issue that clearly warrants further investigation [156,157].

### 4.1. Fatty Acid Synthesis (FAS)

Fatty acid synthesis (FAS) is the production of fatty acids from acetyl CoA and NADPH [158]. This process, which occurs in the cytoplasm, is mediated by the enzymatic activity of fatty acid synthase (FASN). During the initial step of FAS, the ATP citrate lyase ACL (also known as ACLY) converts citrate, an intermediate metabolite from the incomplete TCA cycle, to acetyl CoA and oxaloacetate. Acetyl CoA is a key building block for FAs and cholesterol, and is necessary for post-translational modifications [159]. Acetyl-CoA is converted to malonyl CoA by acetyl-CoA carboxylases (ACCs). Malonyl CoA serves two functions. First malonyl CoA accumulation provides a negative feedback signal to regulate FAO-dependent energy production by suppressing carnitinepalmitoyl transferases (CPTs). Secondly, FASN uses malonyl CoA and acetyl CoA to generate palmitate which serves as a building block for saturated and unsaturated fatty acids [160].

In breast cancer, FAS has been shown to be aberrantly activated through several mechanisms. Notably, FASN and ACL are upregulated in HR+ breast tumors, both have been shown to be essential for cancer cell viability and tumorigenesis and their increased expression in breast tumors is associated with poor prognosis [161]. FASN is required for estrogen-dependent activation of ER signaling, cell growth and survival, and has been shown to contribute to endocrine resistance through AKT/MAPK signaling [162,163,164]. Interestingly, studies have also reported that FASN is activated by SREBP1, which is expressed in response to acquired endocrine resistance, suggesting that the SREBP1/FASN signaling axis may contribute to acquired endocrine therapy resistance in certain subsets of ER+ tumors [165].

Saturated fatty acids, generated by FASN, may provide an inhibitory signal to the FAO pathway. Cancer cells overcome this inhibition by increased expression of stearoyl CoA desaturase (SCD1) which is responsible for converting saturated fatty acids (SFAs) to monounsaturated fatty acids (MUFAs). Interestingly, the content and shift in erythrocyte membrane fatty acids ratios, specifically a decreased SFA to MUFA ratio, which is a characteristic of cancer cells, has been shown to be predictive of cancer status and is responsible for cellular membrane fluidity and invasiveness. Mechanistically, SCD1 expression is regulated by estrogen and to a lesser extent by insulin growth factor 1 (IGF1) through SREBP1c. In breast cancer, SCD1 expression results in increased cellular MUFA levels which are essential for the full activation of AKT signaling and suppression of AMPK activity, resulting in increased cell survival and evasion of apoptotic signals [166,167,168,169,170].

### 4.2. Fatty Acid Oxidation (FAO)

Fatty acid oxidation (FAO) is the mitochondrial process of breaking down long-chain fatty acids to generate FADH2 and NADH as well as acetyl CoA, which subsequently enters the electron transport chain to produce ATP [171]. In this process, long-chain fatty acids (LCFA) from the microenvironment are sensed and transported into the cell through specific receptor and transport proteins including SLC27 family members and CD36 [172,173,174]. Once in the cytoplasm, LCFA are converted to fatty acyl-CoA by the long-chain fatty acid CoA ligase. The carnitine shuttle, which is comprised of CPT1 (carnitine palmitoyltransferase I), ACACT (carnitine/acylcarnitine translocase), and CPT2 (carnitine palmitoyltransferase II) is used to transport acyl-CoA from the cytoplasm across the outer mitochondrial membrane (CPT1), inner mitochondrial membrane space (ACACT) and inner mitochondrial membrane (CPT2). Once across the membrane, carnitine, which is added by CPT1A during the initial step of this process, is recycled to the cytoplasm by CPT2 while fatty acyl CoA is shunted into the mitochondrial matrix where it is oxidized to generate acetyl CoA, FADH2, NADH and ATP.

Emerging data demonstrate that extra-cellular lipids secreted by cancer associated fibroblasts (CAFs) and adipocytes can lead to changes in the tumor microenvironment and drive metabolic reprogramming, including FAO, in neighboring tumor cells. The dynamic metabolic dependency between CAFs and cancer epithelial cells has been shown to promote FAO and inhibit FAS due to decreased transactivation of FASN and FA binding proteins (FABPs) in epithelial cancer cells [145]. Likewise, adipocytes contribute to metabolic reprogramming of tumor cells and enhance their dependency on FAO by providing the required FAs through lipolysis [150,175] and the accompanying increased expression of fatty acid transport protein 1, FATP1/SLC27A1 [174] and the fatty acid receptor, CD36 [176] promoting cancer aggressiveness. In tumors, the presence of white adipose tissue alters the microenvironment by promoting an inflammatory niche and reprogramming metabolism towards increases in lipid utilization and the development of a more aggressive metastatic tumor phenotype [177].

CPT1 and CPT2 family members have been shown to maintain breast cancer cell metabolic plasticity, promote cancer progression and play a key role in resistance to radiation and chemotherapy [178,179,180,181]. Evidence indicates that increased CPT1A expression and subsequently enhanced lipid metabolism occur in response to increased fatty acids available through experimental supplementation, de novo fatty acid synthesis, or through extracellular fatty acids secreted into the microenvironment by adipocytes or CAFs as a result of lipolysis or FAS, respectively [175,182]. While separate studies by Curtis et al. [13] and Gatza et al. [183] determined that CPT1A copy number gains are prevalent in specific subsets of aggressive ER+ tumors, additional evidence has implicated ER [148], AR/androgens [184], miR-107 [185] and miR-328-3p [186] as key regulators of CPT1A expression. Further studies have concluded that in the absence of glucose, the tumor suppressor Snail indirectly induces expression of CPT1-family proteins and FAO. In these tumors, Snail has been shown to inhibit expression of acetyl CoA carboxylase beta (ACC2) which catalyzes the carboxylation of acetyl CoA to malonyl CoA. As such, decreased ACC2 expression inhibits the negative feedback mechanism responsible for regulating CPT1 expression. As a result, decreased ACC2 leads to increased CPT1A levels and activation of FAO [187]. Beyond the direct effects of CPT1A on tumor cells, it was recently reported that CPT1A is secreted by tumor cells in the blood stream and detected as a serum biomarker for breast cancer diagnosis and progression [188]. Although other member of the CPT family and key components of the FAO pathway have been implicated in tumorigenesis in other forms of cancer, including other types of breast cancer [189,190], their role in HR+ breast cancer remains to be determined. Other solute carrier family mediators of the carnitine shuttle might be of interest to investigating in HR+ breast cancer, including OCTN2 and MCT9, given that the expression of these genes is strongly estrogen dependent or correlated with estrogen and/or ER expression [191,192,193]. Due to the advances in our understanding of fatty acid metabolism and the identification aberrant expression of these pathway in HR+ breast cancer, emerging data support FAO, FAS and lipid metabolism as potential therapeutic opportunities; however, it remains unclear why some ER+ tumors may be more dependent on FAS while others appear to require FAO.

## 5. Metabolism in Therapeutic Resistance and Novel Clinical Opportunities

Clinically, HR+ breast tumors can be effectively treated with endocrine-based therapies including aromatase inhibitors (AI), selective estrogen receptor modulators (SERMs), selective ER down-regulators (SERD) as well as gonadotropin-releasing hormone (GnRH) agonists [194]. In addition, radiation and/or chemotherapy are often incorporated into treatment regimens based on tumor burden and/or disease progression and lymph node status [195]. While the majority of patients, in particular those with early stage or low-grade disease, are largely responsive to these treatments, 30–50% of patients will demonstrate de novo or acquired resistance to endocrine therapy leading to disease progression [2,196,197]. As we previously noted, loss of estrogen-dependent growth and/or developed resistance to endocrine-based therapies resulting in increased tumor aggressiveness and progression, including metastasis, has been attributed to *ESR1* mutations, altered MAPK signaling, MYC or transcription factor activation, and/or activation of key growth factors as well as a number of unknown mechanisms [41,198]. Consistent with these data, modified treatment regimens incorporating endocrine-based therapy with targeted inhibitors have increased therapeutic response and limited the onset of resistance. Recent clinical trials have led to the clinical approval of the mTOR inhibitor everolimus in conjunction with exemestane as well as the PI3K inhibitor, alpelisib, in combination with fulvestrant for the treatment of patients with advanced or metastatic ER+, PR+, HER2-negative, PIK3CA mutant tumors [199,200,201]. In addition, the combination of fulvestrant with the AKT inhibitor capivasertib is in clinical trials [202,203,204]. Beyond PI3K/Akt family inhibitors, CDK4/6 inhibitors palbociclib, ribociclib, and abemaciclib have been approved in conjunction with hormone therapy for treatment of HR+/HER2- treatment naïve or hormone therapy treated metastatic breast cancer patients [205,206]. While these novel treatments have shown significant clinical efficacy, their impact is limited to a subset of patients with specific genetic features highlighting the need to continue to develop novel therapeutic approaches to address these challenges.

**Figure 2:**
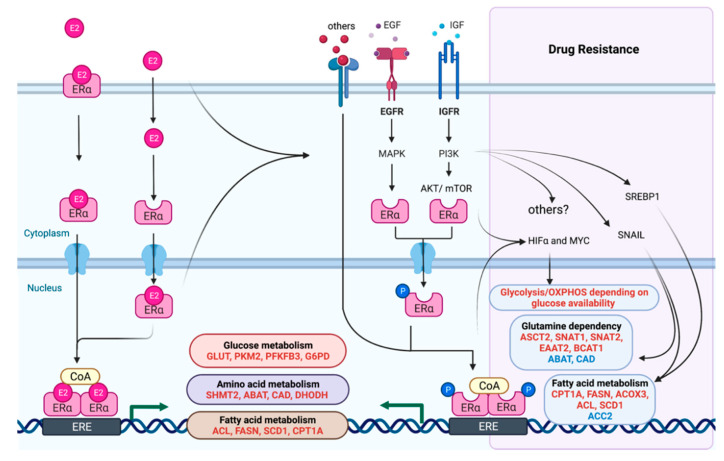
Dynamic changes in major metabolic pathways by ER in sensitive and resistant breast cancer. Upregulated genes are shown in red and downregulated in blue.

Emerging evidence has indicated that metabolic reprogramming regulated by estrogen- and ER-dependent signaling contribute to loss of estrogen-dependent cell growth and therapeutic resistance. As illustrated in Figure 2, changes in glucose, glutamine, amino acid and fatty acid metabolism as well as shifts between metabolic programs due to changing micro-environmental conditions have been shown to contribute to therapeutic sensitivity.

In ER+ breast tumors, a number of studies have reported that increased glycolysis can promote resistance to endocrine-based therapies and can lead to acquired or increased resistance to doxorubicin and paclitaxel [207,208,209]. While no single mechanism has been shown to mediate glycolysis-induced resistance, these studies implicate both direct regulation of glycolysis enzymes and indirect mechanisms promoting glycolysis, possibly estrogen and progesterone, which contribute to altered therapeutic response. In HR+ cell lines, estrogen modulates HIF1α expression through activation of PI3K and mTOR signaling which are upregulated in response to tamoxifen resistance. As cells shift from normal to hypoxic conditions, activation of HIF1α and its cofactor CBP/p300 cooperate to transcriptionally activate down-stream signaling that contributes to cell proliferation, glucose metabolism and chemotherapeutic resistance [210]. Consistent with these findings, estrogen dependent activation of HIF1α is associated with poor patient response and therapeutic resistance in ER+ tumors [211,212,213,214,215,216].

Beyond genetic mechanisms, emerging evidence indicates that breast tumor cells can undergo metabolic reprogramming in response to drug treatment. In a recent study, Goldman and colleagues demonstrated that taxane treatment resulted in metabolic reprogramming of breast tumor cells and was characterized by increased glycolytic and oxidative respiration and glucose flux through the pentose phosphate pathway. Notably this study demonstrated in mouse models that sequential treatment with taxanes, anthracyclines and G6PD inhibitors could enhance survival [217]. Consistent with the potential impact of G6PD and glucose metabolism on acquired resistance, inhibition of 6PGD has been shown to result in increased AMPK signaling which leads to a reduction in ACC1 activity and lipid biosynthesis. As such, inhibiting 6PGD results in cell cycle arrest, alters metabolic reprogramming towards glutamine dependency, and increases chemotherapy sensitivity in breast cancer cells [76,218].

More recently, studies by Lorito and colleagues noted that ER+ cells increased glycolytic metabolism as a result of acquired resistance to the CDK4/6 inhibitor palbociclib [219]. Interestingly, this study reported that differences in glucose dependencies were evident in ER+ cell lines based on HER2 status. The investigators demonstrated that palbociclib sensitive ER^+^/HER2^−^ breast cancer cells are characterized by increased aerobic glycolysis whereas ER^+^/HER2^+^ cells increased glycolytic catabolism as they develop resistance. Consistent with the potential clinical implications of these findings, the investigators demonstrated that targeting glycolysis can re-sensitize resistant ER^+^/HER2^+^ cells to palbociclib and significantly enhance the anti-tumoral effects of the drug in sensitive ER^+^/HER2^−^ cells. While the potential role of HER2 in this study was not clear, additional studies have indicated that increased glycolysis, potentially due to HER2-dependent activation of the glycolytic gene LDHA through HSF1, could promote trastuzumab resistance. Consistent with this premise, HSF1 expression has been shown to be upregulated in trastuzumab resistant ER+ cells, and targeting glycolytic genes, either alone or in combination with trastuzumab, increased their sensitivity to the drug [220] Similarly, overexpressing the glycolytic gene PKM2 decreased MCF7 and T47D sensitivity to chemotherapeutic agents whereas inhibiting glycolysis by 2-DG restored sensitivity [221].

As discussed, amino acid metabolism is essential for regulating key aspects of tumor progression, including metastasis, in advanced HR+ breast cancer. Given the association between increased endocrine therapy resistance and tumor stage and grade, it is not surprising that a number of studies have also linked increased glutaminolysis to therapeutic response. As previously noted, acquired endocrine resistance in ER+ tumors can occur by modulating the amino acid metabolism enzymes including BCAT1, CAD, ABAT, ASCT2, SNAT1 and EAAT2 activity [95,103,104,107,121]. Interestingly, Bacci et al. showed that endocrine resistance downregulated the expression of multiple amino acid transporter genes, but cancer cells compensated for this loss of function, promoted glutamine dependency, and maintained tumor aggressiveness through enhanced autophagy and the upregulation of EAAT2 [222]. Beyond EAAT2, the sodium-dependent neutral amino acid transporter SNAT2, has been shown to mediate endocrine therapy resistance under hypoxic conditions though HIF1α activity [223].

As we previously noted, glutaminolysis can be inhibited through GLS to overcome glutamine dependency in HR+ breast cancer cells that have developed resistance to several therapeutic agents. In response to therapy, GLS is indirectly activated by the loss of the negative feedback mechanisms, including loss of PTEN and RB1, and directly activated by several oncogenes and oncogenic pathways including KRAS, MYC, HIF1α, HIF2α, HER2, JAK/STAT, mTOR and WNT signaling [94,95]. Consistent with these findings, activation of PGC1α/ERRα signaling results in increased glutamine dependency and therapeutic resistance in metastatic breast cancer and circulating cancer cells. Several studies have demonstrated that PGC1α regulates the expression of a necessary gene expression programs, including expression of nuclear export protein exportin 1 (XPO1), that enables tumor cells to maintain metabolic plasticity, increase glutamine dependency, maintain cell growth and sustain invasiveness under limited nutrition conditions [137,138,139,140,141,142]. A number of additional studies have reported that ERRα can promote lapatinib resistance in breast cancer cells through increasing glutamine flux [224].

Consistent with these mechanistic studies, preclinical studies and clinical trials have begun to support glutamine metabolism as a potential therapeutic opportunity in breast cancer. Notably, Demas et al. reported that inhibiting GLS in combination with mTOR enabled HR+ cells to overcome endocrine resistance [95]. While resistance to GLS1-selective inhibitors can occur in some tumors, evidence indicates that concurrent targeting of GLS2 using a pan-glutaminase inhibitor can prevent, or overcome, resistance in preclinical models suggesting the potential clinical impact of this approach [106]. The use of the glutaminase inhibitor, CB-839, in combination with the CDK4/6 inhibitor, palbociclib, resulted in blocking cell cycle progression and improved anti-tumor activity in ER+ breast cancer [225]. In addition to GLS, glutamine dependency can be pharmacologically inhibited by targeting CAD in order to block nucleotide synthesis. Although PALA [N-(phosphonacetyl)-l-aspartate], a pyrimidine nucleotide synthesis and CAD inhibitor, has been shown to sensitize tumor cells to chemotherapy in preclinical models, unfortunately, clinical trials did not support its use for breast cancer due to low efficacy and cytotoxicity [226,227]. In contrast, dihydroorotate dehydrogenase (DHODH) inhibition has shown more promise. Initial clinical studies reported that use of brequinar to inhibit DHODH activity in patients with advanced breast cancer has a moderate favorable impact [228] while a number of more recent preclinical studies suggest that combined DHODH and MEK inhibition may have the potential to overcome chemotherapy resistance [226,229]. Another clinical trial is in progress to evaluate the DHODH inhibitor leflunomide in TNBC patients (ClinicalTrials.gov Identifier: NCT03709446). However, given the association link between altered glutamine metabolism and HR+ breast cancer, it will be interesting to evaluate leflunomide in HR+ preclinical and clinical trials.

A number of studies have provided evidence that fatty acid metabolism, both FAS and FAO, can contribute to therapeutic response and resistance in HR+ breast cancer. Interestingly, however, it remains unclear why some ER+ tumors may be more dependent on FAS while others appear to require FAO. Focusing first on FAS, estrogen-mediated FASN overexpression has been shown to promote tamoxifen resistance through PI3K/AKT/SREBP signaling [163]. In preclinical studies, inhibition of FASN alleviated tamoxifen resistance in ER+/HER+ animal models [230]. Beyond ER-dependent regulation of FASN, PPARα and PPARγ have been shown to contribute to endocrine resistance by modulating FAS. PPARα can mediate FASN phosphorylation and activity to promote endocrine resistance while PPARγ can do so through aberrant activation of Acyl-CoA oxidase 3 (ACOX3) [231,232]. Interestingly, inhibiting the expression of ACLY, ACC, and FASN using the weight loss supplement hydroxycitric acid (HCA) in combination with tamoxifen in preclinical HR+ models resulted in synergistic cytotoxic effects on cell growth [233]. Consistent with mechanistic and preclinical studies suggesting that altered FAS can contribute to the emergence of resistant disease, a number of clinical studies have reported increased efficacy of hormone-based therapies in combination with FAS inhibition. Notably, the FASN selective inhibitor TVB-2640 showed good tolerability and efficiency when combined with taxol in previously treated patients with advanced metastatic breast cancer. In these studies, FASN inhibition resulted in partial regression in ~20% of patients and led to stable disease in the remainder of patients in the study [234].

In clinical studies, the use of proton pump inhibitors (PPIs) including omeprazole and esomeprazole were found to sensitize metastatic breast cancer, including ~72% of HR+ luminal breast cancer patients to chemotherapy [235,236]. PPIs function as a selective FASN inhibitor and have been shown to improve the efficacy of neoadjuvant drugs and to significantly reduce FASN activity in TNBC patients (ClinicalTrials.gov Identifier: NCT02595372) [237]. While these studies were encouraging, the potential benefits of PPI will need to be weighed against the long-term side effects, including cognitive impairment, that has been reported in some breast cancer patients [238]. Interestingly, FASN inhibition was also achievable through dietary modification. Recent work has indicated that supplementing the diet of breast cancer patients with conjugated linoleic acid (CLA) resulted in decreased Ki67 expression in their tumors (ClinicalTrials.gov Identifier: NCT00908791). Earlier mechanistic studies found that this was at least partially due to its negative effect on estrogen receptor signaling [239,240].

In addition to FAS, FAO has been reported to contribute to therapeutic resistance and response in HR+ breast cancer. Notably, CPT1A has been shown to be upregulated and contribute to the development of radiation resistance in ER+ breast tumors and to trastuzumab in HER2+ tumors [178,179]. Since these tumors developed increased dependence on fatty acid metabolism, these data suggest that FAO inhibitors may benefit resistant and metastatic tumors. This idea is supported by studies from other solid tumors where CPT1A expression was associated with metastatic ability, EMT and drug resistant phenotype and its inhibition re-sensitized resistant cells to radiation, hormone therapy and/or and chemo-therapy [180,181,241,242,243,244,245,246]. Mechanistically, endocrine-resistant cells have been shown to express high levels of ERRα/PGC-1β and increased expression of a number of target genes, including CPT1A, which has been shown to increase FAO [247]. Likewise, CD36, which facilitates cellular import of fatty acids from the micro-environment, is upregulated in endocrine-resistant tumors and cell lines. In this study the investigators reported that increased fatty acid import promoted FAO leading to increased cell migration and tamoxifen resistance [248]. While mechanistic, correlative and pre-clinical studies provide intriguing evidence to support FAO as a potential therapeutic target in aggressive ER+ breast tumors, it remains to be determined if these agents can be successfully incorporated into clinical practice for breast cancer given potential hepatocellular toxicities that have been noted with some earlier generation FAO inhibitors [249]. However, recent studies have demonstrated the diabetic drug, metformin, can affect the expression of fatty acid metabolism genes in patient tumor samples and inhibit fatty acid oxidation in breast cancer cell lines. These data suggest that in addition to its noted effect on glucose metabolism metformin may be utilized as a potential FAO inhibitor [250,251]. As such, a number of clinical trials are testing the potential efficacy of this drug in breast cancer patients. A recent phase II trial reported the safety of metformin and moderate efficacy in overweight/obese HR+ breast cancer patients [252]. A conclusion that needs further investigation with a larger study cohort, as this trial was performed on 22 patients. While a number of ongoing trials are evaluating the clinical benefits of metformin (ClinicalTrials.gov Identifier: NCT01042379 and NCT01980823), completed trials suggest that a metformin regimen decreases the incidence of breast cancer for overweight/obese patients with metabolic syndrome who are at risk for this disease [253].

## 6. Conclusions

Breast cancer continues to be a challenging disease to treat due to the high level of molecular heterogeneity between patients which leads to variable clinical responses. This heterogeneity magnifies the importance of developing more precise diagnostic and prognostic tools in order to guide rational therapeutic options in the clinic based on the underlying molecular basis of a patient’s disease. Emerging data now make it clear that metabolic pathways and metabolic plasticity play an essential role in tumor development, progression and therapeutic response. As we have reviewed here, in HR+ breast cancer, these processes are driven, in part, by nuclear receptors, including hormone receptor-dependent signaling. While much of the focus of anti-cancer therapies has been on oncogenic signaling networks, including hormone receptors and kinases, cancer cells show incredible plasticity in rewiring their metabolic preferences according to the available nutrients in order to enhance survival. This plasticity allows tumor cells to navigate evolving environmental conditions in order to maintain cell growth and to survive the metastatic process. Although the exact mechanism and micro-environmental conditions necessary to promote altered metabolism remain to be fully elucidated, this will remain an active area of research given the potential clinical impact of these mechanisms in disease progression and therapeutic response. Importantly, we note that other mechanisms beyond nuclear receptor-mediated processes do promote metabolic reprogramming and contribute to an acquired or inherent resistance mechanism. However due to spatial limitations, these aspects are not discussed in the current report. Regardless, the availability of clinically approved drugs for metabolic diseases which can inhibit multiple metabolic pathways offer the exciting possibility of repurposing these reagents, either alone or as a part of a multi-agent therapeutic regimen, to enhance HR+ breast cancer treatment. Although clinical trials to date examining the efficacy of single agent therapies targeting metabolic regulators have not been successful in advanced breast cancer patients, based on preclinical studies the potential exists that considering metabolic genes and pathways as potential therapeutic opportunities in conjunction with standard-of-care treatments may enhance therapeutic response, limit the emergence of resistance, and minimize the development of residual disease.

## Figures and Tables

**Table 1:** Summary of key metabolic genes regulated by nuclear hormone receptor activity in HR+ breast cancer.

Gene	Mechanism of Regulation
Glucose metabolism	
GLUT 1,3 and 4	ER and PR regulated
G6PD	Regulated by estrogen
LDHA	Regulated by estrogen
PKM2	Regulated by estrogen
PFKP	Partially regulated by ER through SNAIL
Amino acid metabolism	
GLS1	Partially regulated by ER through MYC
GLS2	Partially regulated by ER through GATA3
ASCT2	Partially regulated by ER through MYC
SHMT2	Regulated by ER and through MYC and HIF1α
ABAT	Partially regulated by ER
CAD	Regulated by ER
SNAT2	Partially regulated by ER through HIF1α
SLC7A5	Regulated by ER through LLGL2
Fatty acid metabolism	
ABCA1 and ABCG1	Regulated by estrogen, ER and LXR
SCD1	Regulated by estrogen
OCTN2	Regulated by ER
FASN	Regulated by ER, PPARα and PPARγ
ACOX3	Regulated by PPARγ
CPT1A	Regulated by ER/AR/ERRα

## References

[1] Sung H., Ferlay J., Siegel R.L., Laversanne M., Soerjomataram I., Jemal A., Bray F. (2021). Global cancer statistics 2020: GLOBOCAN estimates of incidence and mortality worldwide for 36 cancers in 185 countries. CA Cancer J. Clin..

[2] Siegel R.L., Miller K.D., Jemal A. (2020). Cancer statistics, 2020. CA Cancer J. Clin..

[3] Yedjou C.G., Sims J.N., Miele L., Noubissi F., Lowe L., Fonseca D.D., Alo R.A., Payton M., Tchounwou P.B. (2019). Health and Racial Disparity in Breast Cancer. Adv. Exp. Med. Biol..

[4] Coughlin S.S. (2019). Epidemiology of Breast Cancer in Women. Adv. Exp. Med. Biol..

[5] Momenimovahed Z., Salehiniya H. (2019). Epidemiological characteristics of and risk factors for breast cancer in the world. Breast Cancer (Dove Med Press).

[6] Forouzanfar M.H., Foreman K.J., Delossantos A.M., Lozano R., Lopez A.D., Murray C.J., Naghavi M. (2011). Breast and cervical cancer in 187 countries between 1980 and 2010: A systematic analysis. Lancet.

[7] Tsang J.Y.S., Tse G.M. (2020). Molecular Classification of Breast Cancer. Adv. Anat. Pathol..

[8] Bastien R.R., Rodriguez-Lescure A., Ebbert M.T., Prat A., Munarriz B., Rowe L., Miller P., Ruiz-Borrego M., Anderson D., Lyons B. (2012). PAM50 breast cancer subtyping by RT-qPCR and concordance with standard clinical molecular markers. BMC Med. Genom..

[9] Ciriello G., Gatza M.L., Beck A.H., Wilkerson M.D., Rhie S.K., Pastore A., Zhang H., McLellan M., Yau C., Kandoth C. (2015). Comprehensive Molecular Portraits of Invasive Lobular Breast Cancer. Cell.

[10] Prat A., Adamo B., Cheang M.C., Anders C.K., Carey L.A., Perou C.M. (2013). Molecular characterization of basal-like and non-basal-like triple-negative breast cancer. Oncologist.

[11] Prat A., Parker J.S., Fan C., Perou C.M. (2012). PAM50 assay and the three-gene model for identifying the major and clinically relevant molecular subtypes of breast cancer. Breast Cancer Res. Treat..

[12] Prat A., Perou C.M. (2011). Deconstructing the molecular portraits of breast cancer. Mol. Oncol.

[13] Curtis C., Shah S.P., Chin S.F., Turashvili G., Rueda O.M., Dunning M.J., Speed D., Lynch A.G., Samarajiwa S., Yuan Y. (2012). The genomic and transcriptomic architecture of 2,000 breast tumours reveals novel subgroups. Nature.

[14] Mertins P., Mani D.R., Ruggles K.V., Gillette M.A., Clauser K.R., Wang P., Wang X., Qiao J.W., Cao S., Petralia F. (2016). Proteogenomics connects somatic mutations to signalling in breast cancer. Nature.

[15] Prat A., Parker J.S., Karginova O., Fan C., Livasy C., Herschkowitz J.I., He X., Perou C.M. (2010). Phenotypic and molecular characterization of the claudin-low intrinsic subtype of breast cancer. Breast Cancer Res..

[16] Waks A.G., Winer E.P. (2019). Breast Cancer Treatment: A Review. JAMA.

[17] Shien T., Iwata H. (2020). Adjuvant and neoadjuvant therapy for breast cancer. Jpn. J. Clin. Oncol..

[18] Ponde N.F., Zardavas D., Piccart M. (2019). Progress in adjuvant systemic therapy for breast cancer. Nat. Rev. Clin. Oncol..

[19] Doan T.B., Graham J.D., Clarke C.L. (2017). Emerging functional roles of nuclear receptors in breast cancer. J. Mol. Endocrinol..

[20] Sever R., Glass C.K. (2013). Signaling by nuclear receptors. Cold Spring Harb. Perspect. Biol..

[21] Lamb C.A., Vanzulli S.I., Lanari C. (2019). Hormone receptors in breast cancer: More than estrogen receptors. Medicina.

[22] Bennesch M.A., Picard D. (2015). Minireview: Tipping the balance: Ligand-independent activation of steroid receptors. Mol. Endocrinol..

[23] Yin D., Wang Y.L., Wang Y.F., Yang L., Zhang L., Tang C., Xie W., Ma Y. (2015). Correlation between Clinical Pathology of Luminal B Breast Cancer and Determination of Estrogen Receptor, Progesterone Receptor and Her2 Expression Combined with Nuclear Morphology. J. Biol. Regul. Homeost. Agents.

[24] Petz L.N., Nardulli A.M. (2000). Sp1 binding sites and an estrogen response element half-site are involved in regulation of the human progesterone receptor A promoter. Mol. Endocrinol..

[25] Khatpe A.S., Adebayo A.K., Herodotou C.A., Kumar B., Nakshatri H. (2021). Nexus between PI3K/AKT and Estrogen Receptor Signaling in Breast Cancer. Cancers.

[26] Lee Y.R., Park J., Yu H.N., Kim J.S., Youn H.J., Jung S.H. (2005). Up-regulation of PI3K/Akt signaling by 17beta-estradiol through activation of estrogen receptor-alpha, but not estrogen receptor-beta, and stimulates cell growth in breast cancer cells. Biochem. Biophys. Res. Commun..

[27] Gelsomino L., Panza S., Giordano C., Barone I., Gu G., Spina E., Catalano S., Fuqua S., Ando S. (2018). Mutations in the estrogen receptor alpha hormone binding domain promote stem cell phenotype through notch activation in breast cancer cell lines. Cancer Lett..

[28] Hao L., Rizzo P., Osipo C., Pannuti A., Wyatt D., Cheung L.W., Sonenshein G., Osborne B.A., Miele L. (2010). Notch-1 activates estrogen receptor-alpha-dependent transcription via IKKalpha in breast cancer cells. Oncogene.

[29] Qi X., Tang J., Loesch M., Pohl N., Alkan S., Chen G. (2006). p38gamma mitogen-activated protein kinase integrates signaling crosstalk between Ras and estrogen receptor to increase breast cancer invasion. Cancer Res..

[30] Yu Y., Hao Y., Feig L.A. (2006). The R-Ras GTPase mediates cross talk between estrogen and insulin signaling in breast cancer cells. Mol. Cell. Biol..

[31] Bostner J., Karlsson E., Pandiyan M.J., Westman H., Skoog L., Fornander T., Nordenskjold B., Stal O. (2013). Activation of Akt, mTOR, and the estrogen receptor as a signature to predict tamoxifen treatment benefit. Breast Cancer Res. Treat..

[32] Soares R., Balogh G., Guo S., Gartner F., Russo J., Schmitt F. (2004). Evidence for the notch signaling pathway on the role of estrogen in angiogenesis. Mol. Endocrinol..

[33] Xiao G., Li X., Li G., Zhang B., Xu C., Qin S., Du N., Wang J., Tang S.C., Zhang J. (2017). MiR-129 blocks estrogen induction of NOTCH signaling activity in breast cancer stem-like cells. Oncotarget.

[34] Kumar S., Srivastav R.K., Wilkes D.W., Ross T., Kim S., Kowalski J., Chatla S., Zhang Q., Nayak A., Guha M. (2019). Estrogen-dependent DLL1-mediated Notch signaling promotes luminal breast cancer. Oncogene.

[35] McGlynn L.M., Tovey S., Bartlett J.M., Doughty J., Cooke T.G., Edwards J. (2013). Interactions between MAP kinase and oestrogen receptor in human breast cancer. Eur. J. Cancer.

[36] Chen J., Sun L. (2012). Formononetin-induced apoptosis by activation of Ras/p38 mitogen-activated protein kinase in estrogen receptor-positive human breast cancer cells. Horm. Metab. Res..

[37] Siersbaek R., Kumar S., Carroll J.S. (2018). Signaling pathways and steroid receptors modulating estrogen receptor alpha function in breast cancer. Genes Dev..

[38] Fuentes N., Silveyra P. (2019). Estrogen receptor signaling mechanisms. Adv. Protein Chem. Struct. Biol..

[39] Ikeda K., Horie-Inoue K., Inoue S. (2015). Identification of estrogen-responsive genes based on the DNA binding properties of estrogen receptors using high-throughput sequencing technology. Acta Pharmacol. Sin..

[40] Cicatiello L., Addeo R., Sasso A., Altucci L., Petrizzi V.B., Borgo R., Cancemi M., Caporali S., Caristi S., Scafoglio C. (2004). Estrogens and progesterone promote persistent CCND1 gene activation during G1 by inducing transcriptional derepression via c-Jun/c-Fos/estrogen receptor (progesterone receptor) complex assembly to a distal regulatory element and recruitment of cyclin D1 to its own gene promoter. Mol. Cell. Biol..

[41] Razavi P., Chang M.T., Xu G., Bandlamudi C., Ross D.S., Vasan N., Cai Y., Bielski C.M., Donoghue M.T.A., Jonsson P. (2018). The Genomic Landscape of Endocrine-Resistant Advanced Breast Cancers. Cancer Cell.

[42] Belachew E.B., Sewasew D.T. (2021). Molecular Mechanisms of Endocrine Resistance in Estrogen-Positive Breast Cancer. Front. Endocrinol..

[43] Nelson C.C., Hendy S.C., Shukin R.J., Cheng H., Bruchovsky N., Koop B.F., Rennie P.S. (1999). Determinants of DNA sequence specificity of the androgen, progesterone, and glucocorticoid receptors: Evidence for differential steroid receptor response elements. Mol. Endocrinol..

[44] Chen C.C., Hardy D.B., Mendelson C.R. (2011). Progesterone receptor inhibits proliferation of human breast cancer cells via induction of MAPK phosphatase 1 (MKP-1/DUSP1). J. Biol. Chem..

[45] Vignon F., Bardon S., Chalbos D., Rochefort H. (1983). Antiestrogenic effect of R5020, a synthetic progestin in human breast cancer cells in culture. J. Clin. Endocrinol. Metab..

[46] Musgrove E.A., Swarbrick A., Lee C.S., Cornish A.L., Sutherland R.L. (1998). Mechanisms of cyclin-dependent kinase inactivation by progestins. Mol. Cell. Biol..

[47] Bardou V.J., Arpino G., Elledge R.M., Osborne C.K., Clark G.M. (2003). Progesterone receptor status significantly improves outcome prediction over estrogen receptor status alone for adjuvant endocrine therapy in two large breast cancer databases. J. Clin. Oncol..

[48] Mohammed H., Russell I.A., Stark R., Rueda O.M., Hickey T.E., Tarulli G.A., Serandour A.A., Birrell S.N., Bruna A., Saadi A. (2015). Progesterone receptor modulates ERalpha action in breast cancer. Nature.

[49] Rajc J., Frohlich I., Mrcela M., Tomas I., Flam J. (2018). Prognostic Impact of Low Estrogen and Progesterone Positivity in Luminal B (Her2 Negative) Breast Cancer. Acta Clin. Croat..

[50] Hu T., Chen Y., Liu Y., Zhang D., Pan J., Long M. (2021). Classification of PR-positive and PR-negative subtypes in ER-positive and HER2-negative breast cancers based on pathway scores. BMC Med. Res. Methodol..

[51] Haughian J.M., Pinto M.P., Harrell J.C., Bliesner B.S., Joensuu K.M., Dye W.W., Sartorius C.A., Tan A.C., Heikkila P., Perou C.M. (2012). Maintenance of hormone responsiveness in luminal breast cancers by suppression of Notch. Proc. Natl. Acad. Sci. USA.

[52] Davey M.G., Ryan E.J., Folan P.J., O’Halloran N., Boland M.R., Barry M.K., Sweeney K.J., Malone C.M., McLaughlin R.J., Kerin M.J. (2021). The impact of progesterone receptor negativity on oncological outcomes in oestrogen-receptor-positive breast cancer. BJS Open.

[53] Kono M., Fujii T., Lim B., Karuturi M.S., Tripathy D., Ueno N.T. (2017). Androgen Receptor Function and Androgen Receptor-Targeted Therapies in Breast Cancer: A Review. JAMA Oncol..

[54] Need E.F., Selth L.A., Harris T.J., Birrell S.N., Tilley W.D., Buchanan G. (2012). Research resource: Interplay between the genomic and transcriptional networks of androgen receptor and estrogen receptor alpha in luminal breast cancer cells. Mol. Endocrinol..

[55] Bleach R., Madden S.F., Hawley J., Charmsaz S., Selli C., Sheehan K.M., Young L.S., Sims A.H., Soucek P., Hill A.D. (2021). Steroid ligands, the forgotten triggers of nuclear receptor action; implications for acquired resistance to endocrine therapy. Clin. Cancer Res..

[56] Noureddine L.M., Tredan O., Hussein N., Badran B., Le Romancer M., Poulard C. (2021). Glucocorticoid Receptor: A Multifaceted Actor in Breast Cancer. Int. J. Mol. Sci..

[57] Conzen S.D. (2008). Minireview: Nuclear receptors and breast cancer. Mol. Endocrinol..

[58] Papi A., Orlandi M. (2016). Role of nuclear receptors in breast cancer stem cells. World J. Stem Cells.

[59] Francis G.A., Fayard E., Picard F., Auwerx J. (2003). Nuclear receptors and the control of metabolism. Annu. Rev. Physiol..

[60] Warburg O. (1956). On the origin of cancer cells. Science.

[61] Gandhi N., Das G.M. (2019). Metabolic Reprogramming in Breast Cancer and Its Therapeutic Implications. Cells.

[62] Jia D., Lu M., Jung K.H., Park J.H., Yu L., Onuchic J.N., Kaipparettu B.A., Levine H. (2019). Elucidating cancer metabolic plasticity by coupling gene regulation with metabolic pathways. Proc. Natl. Acad. Sci. USA.

[63] Wu Z., Wu J., Zhao Q., Fu S., Jin J. (2020). Emerging roles of aerobic glycolysis in breast cancer. Clin. Transl. Oncol..

[64] Vander Heiden M.G., Cantley L.C., Thompson C.B. (2009). Understanding the Warburg effect: The metabolic requirements of cell proliferation. Science.

[65] Godoy A., Ulloa V., Rodriguez F., Reinicke K., Yanez A.J., Garcia Mde L., Medina R.A., Carrasco M., Barberis S., Castro T. (2006). Differential subcellular distribution of glucose transporters GLUT1-6 and GLUT9 in human cancer: Ultrastructural localization of GLUT1 and GLUT5 in breast tumor tissues. J. Cell. Physiol..

[66] Krzeslak A., Wojcik-Krowiranda K., Forma E., Jozwiak P., Romanowicz H., Bienkiewicz A., Brys M. (2012). Expression of GLUT1 and GLUT3 glucose transporters in endometrial and breast cancers. Pathol. Oncol. Res..

[67] Rogers S., Docherty S.E., Slavin J.L., Henderson M.A., Best J.D. (2003). Differential expression of GLUT12 in breast cancer and normal breast tissue. Cancer Lett..

[68] Medina R.A., Meneses A.M., Vera J.C., Guzman C., Nualart F., Astuya A., Garcia M.A., Kato S., Carvajal A., Pinto M. (2003). Estrogen and progesterone up-regulate glucose transporter expression in ZR-75-1 human breast cancer cells. Endocrinology.

[69] Garrido P., Moran J., Alonso A., Gonzalez S., Gonzalez C. (2013). 17beta-estradiol activates glucose uptake via GLUT4 translocation and PI3K/Akt signaling pathway in MCF-7 cells. Endocrinology.

[70] Ko B.H., Paik J.Y., Jung K.H., Lee K.H. (2010). 17beta-estradiol augments 18F-FDG uptake and glycolysis of T47D breast cancer cells via membrane-initiated rapid PI3K-Akt activation. J. Nucl. Med..

[71] Rivenzon-Segal D., Boldin-Adamsky S., Seger D., Seger R., Degani H. (2003). Glycolysis and glucose transporter 1 as markers of response to hormonal therapy in breast cancer. Int. J. Cancer.

[72] Jung K.H., Lee E.J., Park J.W., Lee J.H., Moon S.H., Cho Y.S., Lee K.H. (2019). EGF receptor stimulation shifts breast cancer cell glucose metabolism toward glycolytic flux through PI3 kinase signaling. PLoS ONE.

[73] Stincone A., Prigione A., Cramer T., Wamelink M.M., Campbell K., Cheung E., Olin-Sandoval V., Gruning N.M., Kruger A., Tauqeer Alam M. (2015). The return of metabolism: Biochemistry and physiology of the pentose phosphate pathway. Biol. Rev. Camb. Philos. Soc..

[74] Ge T., Yang J., Zhou S., Wang Y., Li Y., Tong X. (2020). The Role of the Pentose Phosphate Pathway in Diabetes and Cancer. Front. Endocrinol..

[75] Li X.B., Gu J.D., Zhou Q.H. (2015). Review of aerobic glycolysis and its key enzymes—New targets for lung cancer therapy. Thorac. Cancer.

[76] Polat I.H., Tarrado-Castellarnau M., Bharat R., Perarnau J., Benito A., Cortes R., Sabatier P., Cascante M. (2021). Oxidative Pentose Phosphate Pathway Enzyme 6-Phosphogluconate Dehydrogenase Plays a Key Role in Breast Cancer Metabolism. Biology.

[77] Benito A., Polat I.H., Noe V., Ciudad C.J., Marin S., Cascante M. (2017). Glucose-6-phosphate dehydrogenase and transketolase modulate breast cancer cell metabolic reprogramming and correlate with poor patient outcome. Oncotarget.

[78] Mitsuishi Y., Taguchi K., Kawatani Y., Shibata T., Nukiwa T., Aburatani H., Yamamoto M., Motohashi H. (2012). Nrf2 redirects glucose and glutamine into anabolic pathways in metabolic reprogramming. Cancer Cell.

[79] Tarrado-Castellarnau M., de Atauri P., Cascante M. (2016). Oncogenic regulation of tumor metabolic reprogramming. Oncotarget.

[80] Jiang P., Du W., Wang X., Mancuso A., Gao X., Wu M., Yang X. (2011). p53 regulates biosynthesis through direct inactivation of glucose-6-phosphate dehydrogenase. Nat. Cell Biol..

[81] Wairagu P.M., Phan A.N., Kim M.K., Han J., Kim H.W., Choi J.W., Kim K.W., Cha S.K., Park K.H., Jeong Y. (2015). Insulin priming effect on estradiol-induced breast cancer metabolism and growth. Cancer Biol. Ther..

[82] Burke R.E., Harris S.C., McGuire W.L. (1978). Lactate dehydrogenase in estrogen-responsive human breast cancer cells. Cancer Res..

[83] Faubert B., Solmonson A., DeBerardinis R.J. (2020). Metabolic reprogramming and cancer progression. Science.

[84] Fong M.Y., Zhou W., Liu L., Alontaga A.Y., Chandra M., Ashby J., Chow A., O’Connor S.T., Li S., Chin A.R. (2015). Breast-cancer-secreted miR-122 reprograms glucose metabolism in premetastatic niche to promote metastasis. Nat. Cell Biol..

[85] Kim N.H., Cha Y.H., Lee J., Lee S.H., Yang J.H., Yun J.S., Cho E.S., Zhang X., Nam M., Kim N. (2017). Snail reprograms glucose metabolism by repressing phosphofructokinase PFKP allowing cancer cell survival under metabolic stress. Nat. Commun..

[86] Mele L., la Noce M., Paino F., Regad T., Wagner S., Liccardo D., Papaccio G., Lombardi A., Caraglia M., Tirino V. (2019). Glucose-6-phosphate dehydrogenase blockade potentiates tyrosine kinase inhibitor effect on breast cancer cells through autophagy perturbation. J. Exp. Clin. Cancer Res..

[87] Wang M. (2021). Preferential glutamine uptake in cancer cells. Nat. Rev. Nephrol..

[88] Li T., Copeland C., Le A. (2021). Glutamine Metabolism in Cancer. Adv. Exp. Med. Biol..

[89] Bernfeld E., Foster D.A. (2019). Glutamine as an Essential Amino Acid for KRas-Driven Cancer Cells. Trends Endocrinol. Metab..

[90] Li T., Le A. (2018). Glutamine Metabolism in Cancer. Adv. Exp. Med. Biol..

[91] Lampa M., Arlt H., He T., Ospina B., Reeves J., Zhang B., Murtie J., Deng G., Barberis C., Hoffmann D. (2017). Glutaminase is essential for the growth of triple-negative breast cancer cells with a deregulated glutamine metabolism pathway and its suppression synergizes with mTOR inhibition. PLoS ONE.

[92] Ratnikov B., Jeon Y.J., Smith J.W., Ronai Z.A. (2015). Right on TARGET: Glutamine metabolism in cancer. Oncoscience.

[93] Wei Z., Liu X., Cheng C., Yu W., Yi P. (2020). Metabolism of Amino Acids in Cancer. Front. Cell Dev. Biol..

[94] Altman B.J., Stine Z.E., Dang C.V. (2016). From Krebs to clinic: Glutamine metabolism to cancer therapy. Nat. Rev. Cancer.

[95] Demas D.M., Demo S., Fallah Y., Clarke R., Nephew K.P., Althouse S., Sandusky G., He W., Shajahan-Haq A.N. (2019). Glutamine Metabolism Drives Growth in Advanced Hormone Receptor Positive Breast Cancer. Front. Oncol..

[96] Lane A.N., Fan T.W. (2015). Regulation of mammalian nucleotide metabolism and biosynthesis. Nucleic Acids Res..

[97] Aitken S.C., Lippman M.E. (1983). Hormonal regulation of de novo pyrimidine synthesis and utilization in human breast cancer cells in tissue culture. Cancer Res..

[98] Lien E.C., Lyssiotis C.A., Juvekar A., Hu H., Asara J.M., Cantley L.C., Toker A. (2016). Glutathione biosynthesis is a metabolic vulnerability in PI(3)K/Akt-driven breast cancer. Nat. Cell Biol..

[99] Coloff J.L., Murphy J.P., Braun C.R., Harris I.S., Shelton L.M., Kami K., Gygi S.P., Selfors L.M., Brugge J.S. (2016). Differential Glutamate Metabolism in Proliferating and Quiescent Mammary Epithelial Cells. Cell Metab..

[100] Sigoillot F.D., Evans D.R., Guy H.I. (2002). Growth-dependent regulation of mammalian pyrimidine biosynthesis by the protein kinase A and MAPK signaling cascades. J. Biol. Chem..

[101] Zhou Y., Tao L., Zhou X., Zuo Z., Gong J., Liu X., Zhou Y., Liu C., Sang N., Liu H. (2021). DHODH and cancer: Promising prospects to be explored. Cancer Metab..

[102] Wang Y., Bai C., Ruan Y., Liu M., Chu Q., Qiu L., Yang C., Li B. (2019). Coordinative metabolism of glutamine carbon and nitrogen in proliferating cancer cells under hypoxia. Nat. Commun..

[103] Budczies J., Brockmoller S.F., Muller B.M., Barupal D.K., Richter-Ehrenstein C., Kleine-Tebbe A., Griffin J.L., Oresic M., Dietel M., Denkert C. (2013). Comparative metabolomics of estrogen receptor positive and estrogen receptor negative breast cancer: Alterations in glutamine and beta-alanine metabolism. J. Proteom..

[104] Hutschenreuther A., Birkenmeier G., Bigl M., Krohn K., Birkemeyer C. (2013). Glycerophosphoglycerol, Beta-alanine, and pantothenic Acid as metabolic companions of glycolytic activity and cell migration in breast cancer cell lines. Metabolites.

[105] Dias M.M., Adamoski D., Dos Reis L.M., Ascencao C.F.R., de Oliveira K.R.S., Mafra A.C.P., da Silva Bastos A.C., Quintero M., de G Cassago C., Ferreira I.M. (2020). GLS2 is protumorigenic in breast cancers. Oncogene.

[106] Lukey M.J., Cluntun A.A., Katt W.P., Lin M.J., Druso J.E., Ramachandran S., Erickson J.W., Le H.H., Wang Z.E., Blank B. (2019). Liver-Type Glutaminase GLS2 Is a Druggable Metabolic Node in Luminal-Subtype Breast Cancer. Cell Rep..

[107] Khan S., Abdelrahim M., Samudio I., Safe S. (2003). Estrogen receptor/Sp1 complexes are required for induction of cad gene expression by 17beta-estradiol in breast cancer cells. Endocrinology.

[108] Miltenberger R.J., Sukow K.A., Farnham P.J. (1995). An E-box-mediated increase in cad transcription at the G1/S-phase boundary is suppressed by inhibitory c-Myc mutants. Mol. Cell Biol..

[109] Chen K.F., Lai Y.Y., Sun H.S., Tsai S.J. (2005). Transcriptional repression of human cad gene by hypoxia inducible factor-1alpha. Nucleic Acids Res..

[110] Hoffman J.D., Graff R.E., Emami N.C., Tai C.G., Passarelli M.N., Hu D., Huntsman S., Hadley D., Leong L., Majumdar A. (2017). Cis-eQTL-based trans-ethnic meta-analysis reveals novel genes associated with breast cancer risk. PLoS Genet..

[111] Mohamad Fairus A.K., Choudhary B., Hosahalli S., Kavitha N., Shatrah O. (2017). Dihydroorotate dehydrogenase (DHODH) inhibitors affect ATP depletion, endogenous ROS and mediate S-phase arrest in breast cancer cells. Biochimie.

[112] Lieu E.L., Nguyen T., Rhyne S., Kim J. (2020). Amino acids in cancer. Exp. Mol. Med..

[113] Zhang L., Han J. (2017). Branched-chain amino acid transaminase 1 (BCAT1) promotes the growth of breast cancer cells through improving mTOR-mediated mitochondrial biogenesis and function. Biochem. Biophys. Res. Commun..

[114] Tobias D.K., Hazra A., Lawler P.R., Chandler P.D., Chasman D.I., Buring J.E., Lee I.M., Cheng S., Manson J.E., Mora S. (2020). Circulating branched-chain amino acids and long-term risk of obesity-related cancers in women. Sci. Rep..

[115] Ananieva E.A., Wilkinson A.C. (2018). Branched-chain amino acid metabolism in cancer. Curr. Opin. Clin. Nutr. Metab. Care.

[116] Tonjes M., Barbus S., Park Y.J., Wang W., Schlotter M., Lindroth A.M., Pleier S.V., Bai A.H.C., Karra D., Piro R.M. (2013). BCAT1 promotes cell proliferation through amino acid catabolism in gliomas carrying wild-type IDH1. Nat. Med..

[117] Wang P., Wu S., Zeng X., Zhang Y., Zhou Y., Su L., Lin W. (2018). BCAT1 promotes proliferation of endometrial cancer cells through reprogrammed BCAA metabolism. Int. J. Clin. Exp. Pathol..

[118] Xu Y., Yu W., Yang T., Zhang M., Liang C., Cai X., Shao Q. (2018). Overexpression of BCAT1 is a prognostic marker in gastric cancer. Hum. Pathol..

[119] Wang Z.Q., Faddaoui A., Bachvarova M., Plante M., Gregoire J., Renaud M.C., Sebastianelli A., Guillemette C., Gobeil S., Macdonald E. (2015). BCAT1 expression associates with ovarian cancer progression: Possible implications in altered disease metabolism. Oncotarget.

[120] Mayers J.R., Torrence M.E., Danai L.V., Papagiannakopoulos T., Davidson S.M., Bauer M.R., Lau A.N., Ji B.W., Dixit P.D., Hosios A.M. (2016). Tissue of origin dictates branched-chain amino acid metabolism in mutant Kras-driven cancers. Science.

[121] Thewes V., Simon R., Hlevnjak M., Schlotter M., Schroeter P., Schmidt K., Wu Y., Anzeneder T., Wang W., Windisch P. (2017). The branched-chain amino acid transaminase 1 sustains growth of antiestrogen-resistant and ERalpha-negative breast cancer. Oncogene.

[122] Shafei M.A., Flemban A., Daly C., Kendrick P., White P., Dean S., Qualtrough D., Conway M.E. (2021). Differential expression of the BCAT isoforms between breast cancer subtypes. Breast Cancer.

[123] Symonds E.L., Pedersen S.K., Baker R.T., Murray D.H., Gaur S., Cole S.R., Gopalsamy G., Mangira D., LaPointe L.C., Young G.P. (2016). A Blood Test for Methylated BCAT1 and IKZF1 vs. a Fecal Immunochemical Test for Detection of Colorectal Neoplasia. Clin. Transl. Gastroenterol..

[124] Song Y., Zhao B., Xu Y., Ren X., Lin Y., Zhou L., Sun Q. (2020). Prognostic significance of branched-chain amino acid transferase 1 and CD133 in triple-negative breast cancer. BMC Cancer.

[125] Saito Y., Li L., Coyaud E., Luna A., Sander C., Raught B., Asara J.M., Brown M., Muthuswamy S.K. (2019). LLGL2 rescues nutrient stress by promoting leucine uptake in ER(+) breast cancer. Nature.

[126] Ducker G.S., Rabinowitz J.D. (2017). One-Carbon Metabolism in Health and Disease. Cell Metab..

[127] Li A.M., Ducker G.S., Li Y., Seoane J.A., Xiao Y., Melemenidis S., Zhou Y., Liu L., Vanharanta S., Graves E.E. (2020). Metabolic Profiling Reveals a Dependency of Human Metastatic Breast Cancer on Mitochondrial Serine and One-Carbon Unit Metabolism. Mol. Cancer Res..

[128] Yue L., Pei Y., Zhong L., Yang H., Wang Y., Zhang W., Chen N., Zhu Q., Gao J., Zhi M. (2020). Mthfd2 Modulates Mitochondrial Function and DNA Repair to Maintain the Pluripotency of Mouse Stem Cells. Stem Cell Rep..

[129] Tanaka K., Sasayama T., Nagashima H., Irino Y., Takahashi M., Izumi Y., Uno T., Satoh N., Kitta A., Kyotani K. (2021). Glioma cells require one-carbon metabolism to survive glutamine starvation. Acta Neuropathol. Commun..

[130] Shukla K., Singh N., Lewis J.E., Tsang A.W., Boothman D.A., Kemp M.L., Furdui C.M. (2020). MTHFD2 Blockade Enhances the Efficacy of beta-Lapachone Chemotherapy With Ionizing Radiation in Head and Neck Squamous Cell Cancer. Front. Oncol..

[131] Newman A.C., Maddocks O.D.K. (2017). One-carbon metabolism in cancer. Br. J. Cancer.

[132] Koufaris C., Gallage S., Yang T., Lau C.H., Valbuena G.N., Keun H.C. (2016). Suppression of MTHFD2 in MCF-7 Breast Cancer Cells Increases Glycolysis, Dependency on Exogenous Glycine, and Sensitivity to Folate Depletion. J. Proteome Res..

[133] Bernhardt S., Bayerlova M., Vetter M., Wachter A., Mitra D., Hanf V., Lantzsch T., Uleer C., Peschel S., John J. (2017). Proteomic profiling of breast cancer metabolism identifies SHMT2 and ASCT2 as prognostic factors. Breast Cancer Res..

[134] Geeraerts S.L., Kampen K.R., Rinaldi G., Gupta P., Planque M., Louros N., Heylen E., De Cremer K., De Brucker K., Vereecke S. (2021). Repurposing the Antidepressant Sertraline as SHMT Inhibitor to Suppress Serine/Glycine Synthesis-Addicted Breast Tumor Growth. Mol. Cancer Ther..

[135] Li X., Zhang K., Hu Y., Luo N. (2020). ERRalpha activates SHMT2 transcription to enhance the resistance of breast cancer to lapatinib via modulating the mitochondrial metabolic adaption. Biosci. Rep..

[136] Vazquez Rodriguez G., Abrahamsson A., Turkina M.V., Dabrosin C. (2020). Lysine in Combination With Estradiol Promote Dissemination of Estrogen Receptor Positive Breast Cancer via Upregulation of U2AF1 and RPN2 Proteins. Front. Oncol..

[137] Cotul E.K., Zuo Q., Santaliz-Casiano A., Imir O.B., Mogol A.N., Tunc E., Duong K., Lee J.K., Ramesh R., Odukoya E. (2020). Combined Targeting of Estrogen Receptor Alpha and Exportin 1 in Metastatic Breast Cancers. Cancers.

[138] LeBleu V.S., O’Connell J.T., Gonzalez Herrera K.N., Wikman H., Pantel K., Haigis M.C., de Carvalho F.M., Damascena A., Domingos Chinen L.T., Rocha R.M. (2014). PGC-1alpha mediates mitochondrial biogenesis and oxidative phosphorylation in cancer cells to promote metastasis. Nat. Cell Biol..

[139] Sancho P., Burgos-Ramos E., Tavera A., Bou Kheir T., Jagust P., Schoenhals M., Barneda D., Sellers K., Campos-Olivas R., Grana O. (2015). MYC/PGC-1alpha Balance Determines the Metabolic Phenotype and Plasticity of Pancreatic Cancer Stem Cells. Cell Metab..

[140] Tan Z., Luo X., Xiao L., Tang M., Bode A.M., Dong Z., Cao Y. (2016). The Role of PGC1alpha in Cancer Metabolism and its Therapeutic Implications. Mol. Cancer Ther..

[141] Valcarcel-Jimenez L., Macchia A., Crosas-Molist E., Schaub-Clerigue A., Camacho L., Martin-Martin N., Cicogna P., Viera-Bardon C., Fernandez-Ruiz S., Rodriguez-Hernandez I. (2019). PGC1alpha Suppresses Prostate Cancer Cell Invasion through ERRalpha Transcriptional Control. Cancer Res..

[142] Chen E.I., Hewel J., Krueger J.S., Tiraby C., Weber M.R., Kralli A., Becker K., Yates J.R., Felding-Habermann B. (2007). Adaptation of energy metabolism in breast cancer brain metastases. Cancer Res..

[143] Pavlova N.N., Thompson C.B. (2016). The Emerging Hallmarks of Cancer Metabolism. Cell Metab..

[144] Fernandez L.P., Gomez de Cedron M., Ramirez de Molina A. (2020). Alterations of Lipid Metabolism in Cancer: Implications in Prognosis and Treatment. Front. Oncol..

[145] Lopes-Coelho F., Andre S., Felix A., Serpa J. (2018). Breast cancer metabolic cross-talk: Fibroblasts are hubs and breast cancer cells are gatherers of lipids. Mol. Cell. Endocrinol..

[146] Biswas S., Lunec J., Bartlett K. (2012). Non-glucose metabolism in cancer cells—Is it all in the fat?. Cancer Metastasis Rev..

[147] Qu Q., Zeng F., Liu X., Wang Q.J., Deng F. (2016). Fatty acid oxidation and carnitine palmitoyltransferase I: Emerging therapeutic targets in cancer. Cell Death Dis..

[148] Monaco M.E. (2017). Fatty acid metabolism in breast cancer subtypes. Oncotarget.

[149] Cheng C., Geng F., Cheng X., Guo D. (2018). Lipid metabolism reprogramming and its potential targets in cancer. Cancer Commun..

[150] Hultsch S., Kankainen M., Paavolainen L., Kovanen R.M., Ikonen E., Kangaspeska S., Pietiainen V., Kallioniemi O. (2018). Association of tamoxifen resistance and lipid reprogramming in breast cancer. BMC Cancer.

[151] El Roz A., Bard J.M., Huvelin J.M., Nazih H. (2012). LXR agonists and ABCG1-dependent cholesterol efflux in MCF-7 breast cancer cells: Relation to proliferation and apoptosis. Anticancer Res..

[152] Bobin-Dubigeon C., Chauvin A., Brillaud-Meflah V., Boiffard F., Joalland M.P., Bard J.M. (2017). Liver X Receptor (LXR)-regulated Genes of Cholesterol Trafficking and Breast Cancer Severity. Anticancer Res..

[153] Nelson E.R., Wardell S.E., Jasper J.S., Park S., Suchindran S., Howe M.K., Carver N.J., Pillai R.V., Sullivan P.M., Sondhi V. (2013). 27-Hydroxycholesterol links hypercholesterolemia and breast cancer pathophysiology. Science.

[154] Baek A.E., Krawczynska N., Das Gupta A., Dvoretskiy S.V., You S., Park J., Deng Y.H., Sorrells J.E., Smith B.P., Ma L. (2021). The Cholesterol Metabolite 27HC Increases Secretion of Extracellular Vesicles Which Promote Breast Cancer Progression. Endocrinology.

[155] Ghanbari F., Fortier A.M., Park M., Philip A. (2021). Cholesterol-Induced Metabolic Reprogramming in Breast Cancer Cells Is Mediated via the ERRalpha Pathway. Cancers.

[156] Garcia-Estevez L., Moreno-Bueno G. (2019). Updating the role of obesity and cholesterol in breast cancer. Breast Cancer Res..

[157] Dong S., Wang Z., Shen K., Chen X. (2021). Metabolic Syndrome and Breast Cancer: Prevalence, Treatment Response, and Prognosis. Front. Oncol..

[158] Currie E., Schulze A., Zechner R., Walther T.C., Farese R.V. (2013). Cellular fatty acid metabolism and cancer. Cell Metab..

[159] Pietrocola F., Galluzzi L., Bravo-San Pedro J.M., Madeo F., Kroemer G. (2015). Acetyl coenzyme A: A central metabolite and second messenger. Cell Metab..

[160] Rohrig F., Schulze A. (2016). The multifaceted roles of fatty acid synthesis in cancer. Nat. Rev. Cancer.

[161] Hatzivassiliou G., Zhao F., Bauer D.E., Andreadis C., Shaw A.N., Dhanak D., Hingorani S.R., Tuveson D.A., Thompson C.B. (2005). ATP citrate lyase inhibition can suppress tumor cell growth. Cancer Cell.

[162] Menendez J.A., Lupu R. (2017). Fatty acid synthase regulates estrogen receptor-alpha signaling in breast cancer cells. Oncogenesis.

[163] Menendez J.A., Papadimitropoulou A., Vander Steen T., Cuyas E., Oza-Gajera B.P., Verdura S., Espinoza I., Vellon L., Mehmi I., Lupu R. (2021). Fatty Acid Synthase Confers Tamoxifen Resistance to ER+/HER2+ Breast Cancer. Cancers.

[164] Menendez J.A., Oza B.P., Atlas E., Verma V.A., Mehmi I., Lupu R. (2004). Inhibition of tumor-associated fatty acid synthase activity antagonizes estradiol- and tamoxifen-induced agonist transactivation of estrogen receptor (ER) in human endometrial adenocarcinoma cells. Oncogene.

[165] Du T., Sikora M.J., Levine K.M., Tasdemir N., Riggins R.B., Wendell S.G., Van Houten B., Oesterreich S. (2018). Key regulators of lipid metabolism drive endocrine resistance in invasive lobular breast cancer. Breast Cancer Res..

[166] Ferreri C., Sansone A., Ferreri R., Amezaga J., Tueros I. (2020). Fatty Acids and Membrane Lipidomics in Oncology: A Cross-Road of Nutritional, Signaling and Metabolic Pathways. Metabolites.

[167] Belkaid A., Duguay S.R., Ouellette R.J., Surette M.E. (2015). 17beta-estradiol induces stearoyl-CoA desaturase-1 expression in estrogen receptor-positive breast cancer cells. BMC Cancer.

[168] Peck B., Schug Z.T., Zhang Q., Dankworth B., Jones D.T., Smethurst E., Patel R., Mason S., Jiang M., Saunders R. (2016). Inhibition of fatty acid desaturation is detrimental to cancer cell survival in metabolically compromised environments. Cancer Metab..

[169] Angelucci C., D’Alessio A., Iacopino F., Proietti G., Di Leone A., Masetti R., Sica G. (2018). Pivotal role of human stearoyl-CoA desaturases (SCD1 and 5) in breast cancer progression: Oleic acid-based effect of SCD1 on cell migration and a novel pro-cell survival role for SCD5. Oncotarget.

[170] Igal R.A. (2011). Roles of StearoylCoA Desaturase-1 in the Regulation of Cancer Cell Growth, Survival and Tumorigenesis. Cancers.

[171] Carracedo A., Cantley L.C., Pandolfi P.P. (2013). Cancer metabolism: Fatty acid oxidation in the limelight. Nat. Rev. Cancer.

[172] Jay A.G., Hamilton J.A. (2018). The enigmatic membrane fatty acid transporter CD36: New insights into fatty acid binding and their effects on uptake of oxidized LDL. Prostaglandins Leukot. Essent. Fatty Acids.

[173] Yen M.C., Chou S.K., Kan J.Y., Kuo P.L., Hou M.F., Hsu Y.L. (2018). Solute Carrier Family 27 Member 4 (SLC27A4) Enhances Cell Growth, Migration, and Invasion in Breast Cancer Cells. Int. J. Mol. Sci..

[174] Mendes C., Lopes-Coelho F., Ramos C., Martins F., Santos I., Rodrigues A., Silva F., Andre S., Serpa J. (2019). Unraveling FATP1, regulated by ER-beta, as a targeted breast cancer innovative therapy. Sci. Rep..

[175] Balaban S., Shearer R.F., Lee L.S., van Geldermalsen M., Schreuder M., Shtein H.C., Cairns R., Thomas K.C., Fazakerley D.J., Grewal T. (2017). Adipocyte lipolysis links obesity to breast cancer growth: Adipocyte-derived fatty acids drive breast cancer cell proliferation and migration. Cancer Metab..

[176] Zaoui M., Morel M., Ferrand N., Fellahi S., Bastard J.P., Lamaziere A., Larsen A.K., Bereziat V., Atlan M., Sabbah M. (2019). Breast-Associated Adipocytes Secretome Induce Fatty Acid Uptake and Invasiveness in Breast Cancer Cells via CD36 Independently of Body Mass Index, Menopausal Status and Mammary Density. Cancers.

[177] Lengyel E., Makowski L., DiGiovanni J., Kolonin M.G. (2018). Cancer as a Matter of Fat: The Crosstalk between Adipose Tissue and Tumors. Trends Cancer.

[178] Han J., Qu H., Han M., Ding Y., Xie M., Hu J., Chen Y., Dong H. (2021). MSC-induced lncRNA AGAP2-AS1 promotes stemness and trastuzumab resistance through regulating CPT1 expression and fatty acid oxidation in breast cancer. Oncogene.

[179] Han S., Wei R., Zhang X., Jiang N., Fan M., Huang J.H., Xie B., Zhang L., Miao W., Butler A.C. (2019). CPT1A/2-Mediated FAO Enhancement-A Metabolic Target in Radioresistant Breast Cancer. Front. Oncol..

[180] Sung G.J., Choi H.K., Kwak S., Song J.H., Ko H., Yoon H.G., Kang H.B., Choi K.C. (2016). Targeting CPT1A enhances metabolic therapy in human melanoma cells with the BRAF V600E mutation. Int. J. Biochem. Cell Biol..

[181] Tan Z., Xiao L., Tang M., Bai F., Li J., Li L., Shi F., Li N., Li Y., Du Q. (2018). Targeting CPT1A-mediated fatty acid oxidation sensitizes nasopharyngeal carcinoma to radiation therapy. Theranostics.

[182] Qian J., Chen Y., Meng T., Ma L., Meng L., Wang X., Yu T., Zask A., Shen J., Yu K. (2016). Molecular regulation of apoptotic machinery and lipid metabolism by mTORC1/mTORC2 dual inhibitors in preclinical models of HER2+/PIK3CAmut breast cancer. Oncotarget.

[183] Gatza M.L., Silva G.O., Parker J.S., Fan C., Perou C.M. (2014). An integrated genomics approach identifies drivers of proliferation in luminal-subtype human breast cancer. Nat. Genet..

[184] Schlaepfer I.R., Joshi M. (2020). CPT1A-mediated Fat Oxidation, Mechanisms, and Therapeutic Potential. Endocrinology.

[185] Xiong Y., Liu Z., Li Z., Wang S., Shen N., Xin Y., Huang T. (2019). Long noncoding RNA nuclear paraspeckle assembly transcript 1 interacts with microRNA107 to modulate breast cancer growth and metastasis by targeting carnitine palmitoyltransferase1. Int. J. Oncol..

[186] Zeng F., Yao M., Wang Y., Zheng W., Liu S., Hou Z., Cheng X., Sun S., Li T., Zhao H. (2021). Fatty acid beta-oxidation promotes breast cancer stemness and metastasis via the miRNA-328-3p-CPT1A pathway. Cancer Gene Ther..

[187] Yang J.H., Kim N.H., Yun J.S., Cho E.S., Cha Y.H., Cho S.B., Lee S.H., Cha S.Y., Kim S.Y., Choi J. (2020). Snail augments fatty acid oxidation by suppression of mitochondrial ACC2 during cancer progression. Life Sci. Alliance.

[188] Tan Z., Zou Y., Zhu M., Luo Z., Wu T., Zheng C., Xie A., Wang H., Fang S., Liu S. (2021). Carnitine palmitoyl transferase 1A is a novel diagnostic and predictive biomarker for breast cancer. BMC Cancer.

[189] Guan L., Chen Y., Wang Y., Zhang H., Fan S., Gao Y., Jiao T., Fu K., Sun J., Yu A. (2019). Effects of carnitine palmitoyltransferases on cancer cellular senescence. J. Cell. Physiol..

[190] Zaugg K., Yao Y., Reilly P.T., Kannan K., Kiarash R., Mason J., Huang P., Sawyer S.K., Fuerth B., Faubert B. (2011). Carnitine palmitoyltransferase 1C promotes cell survival and tumor growth under conditions of metabolic stress. Genes Dev..

[191] Console L., Scalise M., Mazza T., Pochini L., Galluccio M., Giangregorio N., Tonazzi A., Indiveri C. (2020). Carnitine Traffic in Cells. Link with Cancer. Front. Cell Dev. Biol..

[192] Wang C., Uray I.P., Mazumdar A., Mayer J.A., Brown P.H. (2012). SLC22A5/OCTN2 expression in breast cancer is induced by estrogen via a novel intronic estrogen-response element (ERE). Breast Cancer Res. Treat..

[193] Sohrabi E., Moslemi M., Rezaie E., Nafissi N., Khaledi M., Afkhami H., Fathi J., Zekri A. (2021). The tissue expression of MCT3, MCT8, and MCT9 genes in women with breast cancer. Genes Genom..

[194] Araki K., Miyoshi Y. (2018). Mechanism of resistance to endocrine therapy in breast cancer: The important role of PI3K/Akt/mTOR in estrogen receptor-positive, HER2-negative breast cancer. Breast Cancer.

[195] Harbeck N., Penault-Llorca F., Cortes J., Gnant M., Houssami N., Poortmans P., Ruddy K., Tsang J., Cardoso F. (2019). Breast cancer. Nat. Rev. Dis. Primers.

[196] Musgrove E.A., Sutherland R.L. (2009). Biological determinants of endocrine resistance in breast cancer. Nat. Rev. Cancer.

[197] Clarke R., Tyson J.J., Dixon J.M. (2015). Endocrine resistance in breast cancer—An overview and update. Mol. Cell. Endocrinol..

[198] Lei J.T., Gou X., Seker S., Ellis M.J. (2019). ESR1 alterations and metastasis in estrogen receptor positive breast cancer. J. Cancer Metastasis Treat..

[199] Piccart M., Hortobagyi G.N., Campone M., Pritchard K.I., Lebrun F., Ito Y., Noguchi S., Perez A., Rugo H.S., Deleu I. (2014). Everolimus plus exemestane for hormone-receptor-positive, human epidermal growth factor receptor-2-negative advanced breast cancer: Overall survival results from BOLERO-2dagger. Ann. Oncol..

[200] Jerusalem G., de Boer R.H., Hurvitz S., Yardley D.A., Kovalenko E., Ejlertsen B., Blau S., Ozguroglu M., Landherr L., Ewertz M. (2018). Everolimus Plus Exemestane vs Everolimus or Capecitabine Monotherapy for Estrogen Receptor-Positive, HER2-Negative Advanced Breast Cancer: The BOLERO-6 Randomized Clinical Trial. JAMA Oncol..

[201] Yardley D.A., Noguchi S., Pritchard K.I., Burris H.A., Baselga J., Gnant M., Hortobagyi G.N., Campone M., Pistilli B., Piccart M. (2013). Everolimus plus exemestane in postmenopausal patients with HR(+) breast cancer: BOLERO-2 final progression-free survival analysis. Adv. Ther..

[202] Martorana F., Motta G., Pavone G., Motta L., Stella S., Vitale S.R., Manzella L., Vigneri P. (2021). AKT Inhibitors: New Weapons in the Fight Against Breast Cancer?. Front. Pharmacol..

[203] Vernieri C., Corti F., Nichetti F., Ligorio F., Manglaviti S., Zattarin E., Rea C.G., Capri G., Bianchi G.V., de Braud F. (2020). Everolimus versus alpelisib in advanced hormone receptor-positive HER2-negative breast cancer: Targeting different nodes of the PI3K/AKT/mTORC1 pathway with different clinical implications. Breast Cancer Res..

[204] Narayan P., Prowell T.M., Gao J.J., Fernandes L.L., Li E., Jiang X., Qiu J., Fan J., Song P., Yu J. (2021). FDA Approval Summary: Alpelisib Plus Fulvestrant for Patients with HR-positive, HER2-negative, PIK3CA-mutated, Advanced or Metastatic Breast Cancer. Clin. Cancer Res..

[205] Shah M., Nunes M.R., Stearns V. (2018). CDK4/6 Inhibitors: Game Changers in the Management of Hormone Receptor-Positive Advanced Breast Cancer?. Oncol. Williston Park.

[206] Cardoso F., Senkus E., Costa A., Papadopoulos E., Aapro M., Andre F., Harbeck N., Aguilar Lopez B., Barrios C.H., Bergh J. (2018). 4th ESO-ESMO International Consensus Guidelines for Advanced Breast Cancer (ABC 4)dagger. Ann. Oncol..

[207] Tavares-Valente D., Baltazar F., Moreira R., Queiros O. (2013). Cancer cell bioenergetics and pH regulation influence breast cancer cell resistance to paclitaxel and doxorubicin. J. Bioenerg. Biomembr..

[208] Morandi A., Indraccolo S. (2017). Linking metabolic reprogramming to therapy resistance in cancer. Biochim. Biophys. Acta Rev. Cancer.

[209] He M., Jin Q., Chen C., Liu Y., Ye X., Jiang Y., Ji F., Qian H., Gan D., Yue S. (2019). The miR-186-3p/EREG axis orchestrates tamoxifen resistance and aerobic glycolysis in breast cancer cells. Oncogene.

[210] Semenza G.L. (2012). Hypoxia-inducible factors in physiology and medicine. Cell.

[211] Generali D., Berruti A., Brizzi M.P., Campo L., Bonardi S., Wigfield S., Bersiga A., Allevi G., Milani M., Aguggini S. (2006). Hypoxia-inducible factor-1alpha expression predicts a poor response to primary chemoendocrine therapy and disease-free survival in primary human breast cancer. Clin. Cancer Res..

[212] Yao J., Deng K., Huang J., Zeng R., Zuo J. (2020). Progress in the Understanding of the Mechanism of Tamoxifen Resistance in Breast Cancer. Front. Pharmacol..

[213] Yang J., AlTahan A., Jones D.T., Buffa F.M., Bridges E., Interiano R.B., Qu C., Vogt N., Li J.L., Baban D. (2015). Estrogen receptor-alpha directly regulates the hypoxia-inducible factor 1 pathway associated with antiestrogen response in breast cancer. Proc. Natl. Acad. Sci. USA.

[214] Sudhagar S., Sathya S., Lakshmi B.S. (2011). Rapid non-genomic signalling by 17beta-oestradiol through c-Src involves mTOR-dependent expression of HIF-1alpha in breast cancer cells. Br. J. Cancer.

[215] Surazynski A., Miltyk W., Prokop I., Palka J. (2013). The effect of estrogen on prolidase-dependent regulation of HIF-1alpha expression in breast cancer cells. Mol. Cell. Biochem..

[216] George A.L., Rajoria S., Suriano R., Mittleman A., Tiwari R.K. (2012). Hypoxia and estrogen are functionally equivalent in breast cancer-endothelial cell interdependence. Mol. Cancer.

[217] Goldman A., Khiste S., Freinkman E., Dhawan A., Majumder B., Mondal J., Pinkerton A.B., Eton E., Medhi R., Chandrasekar V. (2019). Targeting tumor phenotypic plasticity and metabolic remodeling in adaptive cross-drug tolerance. Sci. Signal..

[218] Yang X., Peng X., Huang J. (2018). Inhibiting 6-phosphogluconate dehydrogenase selectively targets breast cancer through AMPK activation. Clin. Transl. Oncol..

[219] Lorito N., Bacci M., Smiriglia A., Mannelli M., Parri M., Comito G., Ippolito L., Giannoni E., Bonechi M., Benelli M. (2020). Glucose Metabolic Reprogramming of ER Breast Cancer in Acquired Resistance to the CDK4/6 Inhibitor Palbociclib(+). Cells.

[220] Zhao Y., Liu H., Liu Z., Ding Y., Ledoux S.P., Wilson G.L., Voellmy R., Lin Y., Lin W., Nahta R. (2011). Overcoming trastuzumab resistance in breast cancer by targeting dysregulated glucose metabolism. Cancer Res..

[221] Qian Y., Bi L., Yang Y., Wang D. (2018). Effect of pyruvate kinase M2-regulating aerobic glycolysis on chemotherapy resistance of estrogen receptor-positive breast cancer. Anticancer Drugs.

[222] Bacci M., Lorito N., Ippolito L., Ramazzotti M., Luti S., Romagnoli S., Parri M., Bianchini F., Cappellesso F., Virga F. (2019). Reprogramming of Amino Acid Transporters to Support Aspartate and Glutamate Dependency Sustains Endocrine Resistance in Breast Cancer. Cell Rep..

[223] Morotti M., Bridges E., Valli A., Choudhry H., Sheldon H., Wigfield S., Gray N., Zois C.E., Grimm F., Jones D. (2019). Hypoxia-induced switch in SNAT2/SLC38A2 regulation generates endocrine resistance in breast cancer. Proc. Natl. Acad. Sci. USA.

[224] Deblois G., Smith H.W., Tam I.S., Gravel S.P., Caron M., Savage P., Labbe D.P., Begin L.R., Tremblay M.L., Park M. (2016). ERRalpha mediates metabolic adaptations driving lapatinib resistance in breast cancer. Nat. Commun..

[225] Emberley E., Bennett M. (2018). The glutaminase inhibitor CB-839 synergizes with CDK4/6 and PARP inhibitors in pre-clinical tumor models. Cancer Res..

[226] Brown K.K., Spinelli J.B., Asara J.M., Toker A. (2017). Adaptive Reprogramming of De Novo Pyrimidine Synthesis Is a Metabolic Vulnerability in Triple-Negative Breast Cancer. Cancer Discov..

[227] Taylor S.G.T., Davis T.E., Falkson G., Keller A.M. (1982). PALA in advanced breast cancer. A phase II pilot study by the ECOG. Am. J. Clin. Oncol..

[228] Cody R., Stewart D., DeForni M., Moore M., Dallaire B., Azarnia N., Gyves J. (1993). Multicenter phase II study of brequinar sodium in patients with advanced breast cancer. Am. J. Clin. Oncol..

[229] Koundinya M., Sudhalter J., Courjaud A., Lionne B., Touyer G., Bonnet L., Menguy I., Schreiber I., Perrault C., Vougier S. (2018). Dependence on the Pyrimidine Biosynthetic Enzyme DHODH Is a Synthetic Lethal Vulnerability in Mutant KRAS-Driven Cancers. Cell Chem. Biol..

[230] Menendez J.A., Mehmi I., Papadimitropoulou A., Vander Steen T., Cuyas E., Verdura S., Espinoza I., Vellon L., Atlas E., Lupu R. (2020). Fatty Acid Synthase Is a Key Enabler for Endocrine Resistance in Heregulin-Overexpressing Luminal B-Like Breast Cancer. Int. J. Mol. Sci..

[231] Fan P., Abderrahman B., Chai T.S., Yerrum S., Jordan V.C. (2018). Targeting Peroxisome Proliferator-Activated Receptor gamma to Increase Estrogen-Induced Apoptosis in Estrogen-Deprived Breast Cancer Cells. Mol. Cancer Ther..

[232] Chandran K., Goswami S., Sharma-Walia N. (2016). Implications of a peroxisome proliferator-activated receptor alpha (PPARalpha) ligand clofibrate in breast cancer. Oncotarget.

[233] Ismail A., Doghish A.S., Elsadek B.E., Salama S.A., Mariee A.D. (2020). Hydroxycitric acid potentiates the cytotoxic effect of tamoxifen in MCF-7 breast cancer cells through inhibition of ATP citrate lyase. Steroids.

[234] Falchook G., Infante J., Arkenau H.T., Patel M.R., Dean E., Borazanci E., Brenner A., Cook N., Lopez J., Pant S. (2021). First-in-human study of the safety, pharmacokinetics, and pharmacodynamics of first-in-class fatty acid synthase inhibitor TVB-2640 alone and with a taxane in advanced tumors. EClinicalMedicine.

[235] Wang B.Y., Zhang J., Wang J.L., Sun S., Wang Z.H., Wang L.P., Zhang Q.L., Lv F.F., Cao E.Y., Shao Z.M. (2015). Intermittent high dose proton pump inhibitor enhances the antitumor effects of chemotherapy in metastatic breast cancer. J. Exp. Clin. Cancer Res..

[236] Goh W., Sleptsova-Freidrich I., Petrovic N. (2014). Use of proton pump inhibitors as adjunct treatment for triple-negative breast cancers. An introductory study. J. Pharm. Pharm. Sci..

[237] Sardesai S.D., Thomas A., Gallagher C., Lynce F., Ottaviano Y.L., Ballinger T.J., Schneider B.P., Storniolo A.M., Bauchle A., Althouse S.K. (2021). Inhibiting Fatty Acid Synthase with Omeprazole to Improve Efficacy of Neoadjuvant Chemotherapy in patients with Operable TNBC. Clin. Cancer Res..

[238] Madison A.A., Woody A., Bailey B., Lustberg M.B., Ramaswamy B., Wesolowski R., Williams N., Reinbolt R., VanDeusen J.B., Sardesai S. (2020). Cognitive problems of breast cancer survivors on proton pump inhibitors. J. Cancer Surviv..

[239] Tanmahasamut P., Liu J., Hendry L.B., Sidell N. (2004). Conjugated linoleic acid blocks estrogen signaling in human breast cancer cells. J. Nutr..

[240] Liu J., Sidell N. (2005). Anti-estrogenic effects of conjugated linoleic acid through modulation of estrogen receptor phosphorylation. Breast Cancer Res. Treat..

[241] Nimmakayala R.K., Leon F., Rachagani S., Rauth S., Nallasamy P., Marimuthu S., Shailendra G.K., Chhonker Y.S., Chugh S., Chirravuri R. (2021). Metabolic programming of distinct cancer stem cells promotes metastasis of pancreatic ductal adenocarcinoma. Oncogene.

[242] Schlaepfer I.R., Rider L., Rodrigues L.U., Gijón M.A., Pac C.T., Romero L., Cimic A., Sirintrapun S.J., Glodé L.M., Eckel R.H. (2014). Lipid catabolism via CPT1 as a therapeutic target for prostate cancer. Mol. Cancer Ther..

[243] Ren M., Xu H., Xia H., Tang Q., Bi F. (2021). Simultaneously targeting SOAT1 and CPT1A ameliorates hepatocellular carcinoma by disrupting lipid homeostasis. Cell Death Discov..

[244] Ricciardi M.R., Mirabilii S., Allegretti M., Licchetta R., Calarco A., Torrisi M.R., Foa R., Nicolai R., Peluso G., Tafuri A. (2015). Targeting the leukemia cell metabolism by the CPT1a inhibition: Functional preclinical effects in leukemias. Blood.

[245] Sawyer B.T., Qamar L., Yamamoto T.M., McMellen A., Watson Z.L., Richer J.K., Behbakht K., Schlaepfer I.R., Bitler B.G. (2020). Targeting Fatty Acid Oxidation to Promote Anoikis and Inhibit Ovarian Cancer Progression. Mol. Cancer Res..

[246] Flaig T.W., Salzmann-Sullivan M., Su L.J., Zhang Z., Joshi M., Gijon M.A., Kim J., Arcaroli J.J., Van Bokhoven A., Lucia M.S. (2017). Lipid catabolism inhibition sensitizes prostate cancer cells to antiandrogen blockade. Oncotarget.

[247] Duan L., Calhoun S., Shim D., Perez R.E., Blatter L.A., Maki C.G. (2021). Fatty acid oxidation and autophagy promote endoxifen resistance and counter the effect of AKT inhibition in ER-positive breast cancer cells. J. Mol. Cell Biol..

[248] Liang Y., Han H., Liu L., Duan Y., Yang X., Ma C., Zhu Y., Han J., Li X., Chen Y. (2018). CD36 plays a critical role in proliferation, migration and tamoxifen-inhibited growth of ER-positive breast cancer cells. Oncogenesis.

[249] Wang J., Li Y. (2019). CD36 tango in cancer: Signaling pathways and functions. Theranostics.

[250] Lord S.R., Collins J.M., Cheng W.C., Haider S., Wigfield S., Gaude E., Fielding B.A., Pinnick K.E., Harjes U., Segaran A. (2020). Transcriptomic analysis of human primary breast cancer identifies fatty acid oxidation as a target for metformin. Br. J. Cancer.

[251] Roshan M.H., Shing Y.K., Pace N.P. (2019). Metformin as an adjuvant in breast cancer treatment. SAGE Open Med..

[252] Yam C., Esteva F.J., Patel M.M., Raghavendra A.S., Ueno N.T., Moulder S.L., Hess K.R., Shroff G.S., Hodge S., Koenig K.H. (2019). Efficacy and safety of the combination of metformin, everolimus and exemestane in overweight and obese postmenopausal patients with metastatic, hormone receptor-positive, HER2-negative breast cancer: A phase II study. Invest. New Drugs.

[253] Tapia E., Villa-Guillen D.E., Chalasani P., Centuori S., Roe D.J., Guillen-Rodriguez J., Huang C., Galons J.P., Thomson C.A., Altbach M. (2021). A randomized controlled trial of metformin in women with components of metabolic syndrome: Intervention feasibility and effects on adiposity and breast density. Breast Cancer Res. Treat..

